# Short-term effects of temperature-related indices on emergency ambulance dispatches due to mental and behavioral disorders in Shenzhen, China

**DOI:** 10.3389/fpubh.2024.1343550

**Published:** 2024-05-30

**Authors:** Ziming Yin, Maidina Jingesi, Zhao Yin, Siyi Chen, Suli Huang, Jinquan Cheng, Xiaoheng Li, Ning Liu, Peng Wang, Ping Yin, Hongwei Jiang

**Affiliations:** ^1^Department of Epidemiology and Biostatistics, School of Public Health, Tongji Medical College, Huazhong University of Science and Technology, Wuhan, China; ^2^Department of Pharmacy, The First Affiliated Hospital of Zhengzhou University, Zhengzhou, China; ^3^Shenzhen Center for Disease Control and Prevention, Shenzhen, China; ^4^Department of Environment and Health, Shenzhen Center for Disease Control and Prevention, Shenzhen, China

**Keywords:** emergency ambulance dispatches, mental and behavioral disorders, temperature related indices, humidity index, distributed lag non-linear models

## Abstract

**Introduction:**

The precise associations between temperature-related indices and mental and behavioral disorders (MBDs) have yet to be fully elucidated. Our study aims to ascertain the most effective temperature-related index and assess its immediate impact on emergency ambulance dispatches (EADs) due to MBDs in Shenzhen, China.

**Methods:**

EADs data and meteorological data from January 1, 2013, to December 31, 2020, in Shenzhen were collected. Distributed lag non-linear models (DLNMs) were utilized to examine the non-linear and lagged effects of temperature-related indices on EADs due to MBDs. The Quasi Akaike Information criterion (QAIC) was used to determine the optimal index after standardizing temperature-related indices. After adjusting for confounding factors in the model, we estimated the immediate and cumulative effects of temperature on EADs due to MBDs.

**Results:**

The analysis of short-term temperature effects on EADs due to MBDs revealed Humidex as the most suitable index. Referring to the optimal Humidex (3.2^th^ percentile, 12.00°C), we observed a significant effect of Humidex over the threshold (34.6^th^ percentile, 26.80°C) on EADs due to MBDs at lag 0–5. The cumulative relative risks for high temperature (90^th^ percentile, 41.90°C) and extreme high temperature (99^th^ percentile, 44.20°C) at lag 0–5 were 1.318 (95% CI: 1.159–1.499) and 1.338 (95% CI: 1.153–1.553), respectively. No significant cold effect was observed on EADs due to MBDs.

**Conclusion:**

High Humidex was associated with more EADs due to MBDs in subtropical regions. Health authorities should implement effective measures to raise public awareness of risks related to high temperature and protect vulnerable populations.

## 1 Introduction

Mental and behavioral disorders (MBDs) are serious mental health conditions characterized by changes in cognition, emotion, and behavior. These disorders often cause distress and functional impairment, including mood disorders, neurotic disorders, schizophrenia, organic mental disorders, and others. The World Health Organization estimated that approximately 970 million individuals worldwide had a mental disorder in 2019 (1). A global prevalence study of MBDs reported that 29.2% of respondents had experienced at least one MBDs in their lifetime (2). Another study found that MBDs accounted for 13.0% of global disability-adjusted life years (DALYs) (3). DALYs, a metric utilized to evaluate the global impact of a disease or health condition, take into consideration both the years of life lost due to premature mortality and the years of life lived with disability. It is important to note that as the disease burden escalates, the value of DALYs also rises. In China, the lifetime weighted prevalence of MBDs (excluding dementia) was 16.6% among individuals (4). Furthermore, a systematic analysis conducted in 2019 indicated that the age-standardized DALYs per 100,000 due to MBDs ranged from 1150.2 to 1409.1 in China (5).

Climate change has resulted in a wide range of extreme weather events globally, including droughts, rainstorms, heat waves, cold spells, and so forth (6). These extreme weather events have had unprecedented global impacts, leading to an increase in morbidity and mortality (7). The relationship between climate change and MBDs has recently garnered increased attention. Some studies have revealed that climate change may have a direct or indirect long-term impact on MBDs (8–10). Researches conducted in Quebec and Toronto found a correlation between daily mean temperature and an increase in emergency room visits due to MBDs (11, 12). A study in England indicated that for every 1°C increase in temperature above the 93^rd^ percentile of the yearly temperature range, the mortality of individuals with MBDs increased by 4.9% (13). Similarly, a study in Shanghai demonstrated that compared to the median temperature (18.30°C), the relative risk (RR) of extreme high temperature (33.10°C, 99^th^ percentile) on MBDs was 1.266 (95% CI: 1.074–1.493) at lag 0–1 (14). Additionally, Niu et al. found that both high and low apparent temperatures could be risk factors for psychiatric emergency room visits (15).

While several studies have explored the relationship between ambient temperature and MBDs (13, 16, 17), few have considered a composite index that combines temperature with other meteorological factors. Besides temperature, other environmental factors, such as humidity and sunshine hours, may also influence the occurrence of mental disorders. A study conducted in New York State found that the risk of mental illnesses peaked during hot and humid conditions, particularly when there were high levels of sun radiation, relative humidity, and temperature simultaneously (18). Taniguchi et al. demonstrated that exposure to sunlight positively affects emotional and psychological health (19). Some studies have examined the relationships between weather variables and health using comprehensive indices. For example, a cross-sectional study in Italy used the humidex index as a predictor of involuntary admission in psychosis (20). Furthermore, comprehensive temperature indices such as physically equivalent temperature have also been employed to explore the links between temperature and MBDs (21). However, despite the use of composite indices in these studies, only a single index was used without any comparison between different composite indices.

Our study aims to investigate the relationships between ten commonly used temperature-related indices and emergency ambulance dispatches (EADs) due to MBDs in Shenzhen. We utilized a standardization methodology to address the scale differences among various composite indices, enabling a more straightforward comparison of models and ultimately identifying the optimal index. Our main focus is to examine low and high temperature trigger acute MBDs attacks in Shenzhen and the lagged effect of such associations. This research holds significant value for the study of MBDs in subtropical regions. By identifying the meteorological factors contributing to an increase in EADs due to MBDs, we seek to uncover the short-term and lagged impacts of specific meteorological conditions on acute episodes in MBDs patients. This enhances our understanding of health risks for MBDs patients under different meteorological conditions, providing a scientific basis for the formulation of corresponding public health policies.

## 2 Materials and methods

### 2.1 Research area

This research was conducted in Shenzhen (N22°27′–22°52′, E113°46′–114°37′), a coastal city in southern China. As of the end of 2020, Shenzhen encompasses an area of 1997.47 km^2^ with a permanent population of 17.63 million. The city is situated at a low latitude and experiences a subtropical monsoon climate with mild temperatures and abundant rainfall and sunshine. Historical meteorological data shows an annual average temperature of 23.00°C, with the highest recorded temperature at 37.60°C and the lowest at 1.90°C (22).

### 2.2 Data collection

The data collected for this study spanned from 2013 to 2020 in Shenzhen and included EADs data, meteorological data, and air pollutants data. EADs data, containing dispatch details such as date, time, and cause, as well as patient information like patient ID, age, gender, address, symptoms, chief complaints, initial diagnosis, and supplementary diagnosis, were provided by the Shenzhen First-aid Command Center. MBDs were defined based on the 10th Revision of the International Classification of Diseases (ICD-10), with disease codes ranging from F01 to F99. We conducted data screening according to strict diagnostic criteria, and only included cases where both the initial and supplementary diagnoses indicate mental and behavioral disorders. Daily count data were obtained by counting the number of MBD cases per day. Daily meteorological data, including mean temperature (Tmean, °C), minimum temperature (Tmin, °C), maximum temperature (Tmax, °C), relative humidity (RH, %), and wind speed (WS, m/s), were obtained from the Shenzhen Meteorological Service Center. Air pollutants variables comprised particulate matter less than 2.5 μm in aerodynamic diameter (PM_2.5_, μg/m^3^), particulate matter less than 10 μm in aerodynamic diameter (PM_10_, μg/m^3^), nitrogen dioxide (NO_2_, μg/m^3^), sulfur dioxide (SO_2_, μg/m^3^), ozone (O_3_, μg/m^3^), and carbon monoxide (CO, mg/m^3^). Daily levels of air pollutants were determined by averaging measurements from seven (eleven since 2017) standard monitoring stations. To ensure the quality of environmental data, both air pollutants and meteorological variables were monitored in accordance with the quality assurance and quality control (QA/QC) processes established by the China Meteorological Bureau and China Environmental Protection Administration.

### 2.3 Temperature-related indices

Our study incorporated 10 commonly used temperature-related indices to examine the associations between meteorological factors and MBDs. Supplementary Table S1 presents a detailed description of these indices, including the daily mean, minimum, and maximum temperature (Tmean, Tmin, and Tmax), apparent temperature (AT) (23), Rothfusz’s heat index (RHI) (24), wind chill index (WCI) (25), effective temperature (ET) (26), net effective temperature (NET) (25), humidity index (Humidex) (27), and alternative temperature-humidity index (THIa) (28).

### 2.4 Statistical analysis

We utilized the distributed lag non-linear models (DLNMs) and Quasi Akaike Information Criterion (QAIC) to determine the most suitable index from 10 temperature-related comprehensive indices. Subsequently, we assessed the impact of the optimal index on EADs due to MBDs and conducted subgroup analysis. To account for the non-linear and lagged effect of the relationships between meteorological variables and mental health (12, 29), we employed DLNMs in conjunction with a quasi-Poisson regression model to analyze the associations between temperature-related indices (both raw measured values and their standardized values—Z score) and EADs due to MBDs. DLNMs integrate prediction and lagged effect into a cross-basis, which is a two-dimensional matrix facilitating the examination of lag-exposure-response effects over specific lag periods (30, 31). We standardize the temperature-related index X*_i, t_* (*i* = 1–10 for ten temperature-related indices) on the day (*t*) into a standardized value (Z*_i, t_*) to eliminate the influence of different units and scales. The equation was as follows:


$$Z_{i,t}=\frac{X_{i,t}-\mu_{i}}{\sigma_{i}}$$

Where *μ_i_* is the mean and *σ_i_* is the standard deviation of ten temperature-related indices. The DLNMs for EADs due to MBDs at day (*t*) are presented as follows:


$$Y_{t}\sim \mathrm{Quasi}-\mathrm{Poisson}\;\left(\mu_{t}\right)$$

$$\begin{array}{l} \log\left(\mu_{t}\right)=\alpha +cb_{t,l}+\mathrm{ns}\left(RH\right)+\mathrm{ns}\left(WS\right)+\beta \cdot \mathrm{pollutant}+\\ \;\mathrm{ns}\left(\mathrm{time}\right)+\lambda \cdot \mathrm{holida}y_{t}+\gamma \cdot \mathrm{do}w_{t}\text{.} \end{array}$$

Where *Y_t_* represents the count of EADs due to MBDs at day *t*, and *μ_t_* is the expected number of EADs due to MBDs at day *t*; *α* refers to the intercept; $c{b_{t}}_{,l}$ represents a cross-basis object generated by DLNMs, which is used to estimate the non-linear and lagged effects of temperature-related indices. The *l* denotes the lag days. Based on previous studies, we chose a maximum lag of 14 days (32). We also conducted sensitivity analyses to evaluate the cumulative effects from lag 0–7 to lag 0–21 (14, 29). We applied a natural cubic spline with 3 degrees of freedom (*df*) for both temperature-related indices and lag space to construct the final model (23). Furthermore, we used natural cubic spline functions, *ns*(*RH*) and *ns*(*WS*), for relative humidity and wind speed, respectively, and assigned 3 *df* to each of them to control for their potential confounding effects (23). The long-term trend was modeled by a natural cubic spline function of time, *ns*(*time*), with 4 *df* per year, determined by the QAIC value and Partial autocorrelation coefficient (PACF) for *ns*(*time*), as shown in Supplementary Figure S1. QAIC considers the model’s goodness of fit and complexity to prevent overfitting by penalizing over-parameterization. Generally, a smaller QAIC value indicates a better fit for the model. PACF is the sum of the absolute values of the partial autocorrelation function of the residuals, used to compare the autocorrelation of residuals between different models. A lower PACF value typically signifies a lower level of autocorrelation in the residuals. Additionally, the variables *pollutant*, *holiday_t_,* and *dow_t_* represent air pollutant levels, public holidays (33), and day of week, respectively, with *β*, *λ*, and *γ* as the corresponding regression coefficients.

Notably, owing to the strong correlation between meteorological factors and air pollutants, with correlation coefficients exceeding 0.6 (Supplementary Figure S2), the final models included only *RH*, *WS*, and *NO_2_* to avoid potential biases resulting from multicollinearity, as was undertaken in a prior study (29). It is important to highlight that either *RH* or *WS* would be excluded from the model when the temperature-related indices incorporated relative humidity or wind speed. Furthermore, NO_2_ was directly incorporated into the model as a linear function, in accordance with previous research suggesting a linear association between NO_2_ and mental health (34). For these ten temperature-related indices, estimating the risk of low and high temperature effects using the same measurement method is not feasible. Therefore, we calculated the cumulative RRs using the standardized Z values for each index (34). Subsequently, we used QAIC to select the optimal temperature-related index. RRs and corresponding 95% confidence intervals (CI) for EADs due to MBDs were then computed at the 1^st^, 10^th^, 90^th^, and 99^th^ percentiles of the optimal temperature-related index, assessing the effect of extreme low temperature, low temperature, high temperature, and extreme high temperature on EADs due to MBDs.

To identify the vulnerable population, we performed subgroup analysis based on sex (male or female) and age (0–14, 15–39, 40–59, and 60+). We also employed a stratified approach to examine whether temperature-related indices affect EADs due to MBDs differently across seasons (35). Given that Shenzhen is located at a relatively low latitude and experiences a subtropical monsoon climate with a long summer and short winter, the traditional astronomical four-season method may not accurately reflect the city’s climatic characteristics. Hence, following the seasonal classification method provided by the Shenzhen Meteorological Bureau (36), we categorized the data into warm (from April 20th to November 7th) and cold seasons (remaining days), conducting separate analyses for each (37). Furthermore, we conducted sensitivity analyses to assess the robustness of our model. These analyses included the following modifications: (1) dividing the data into two periods: the first four years (2013–2016) and the last four years (2017–2020) to investigate the impact of temporal change on the results; (2) altering the *df* (2, 4, 5) for wind speed; (3) adjusting the *df* (5–8) for long-term trend; (4) modifying the lag days (7–21 days) for daily temperature-related indices; (5) substituting NO_2_ confounding factors with other pollutants confounding factors; (6) according to the Lancet’s paper and the official announcement of the onset date of the first COVID-19 patient (38), omitting data after December 1, 2019 to examine the potential impact of the COVID-19 epidemic on the results.

In our study, we utilized a two-dimensional smooth response surface model to investigate the interaction effect between air pollutants and meteorological variables on EADs due to MBDs. It models the joint effect of air pollutants and meteorological variables on EADs due to MBDs as a continuous function of both variables. The model was as follows:


$$\begin{array}{l} \log\left(\mu_{t}\right)=\alpha +Te\left(\mathrm{air}\;\mathrm{pollutants}:\mathrm{meteorological}\;\mathrm{variables}\right)+\\ \;\mathrm{ns}\left(RH\right)+\mathrm{ns}\left(WS\right)+\mathrm{ns}\left(\mathrm{time}\right)+\lambda \cdot \mathrm{holida}y_{t}+\gamma \cdot \mathrm{do}w_{t}\text{.} \end{array}$$

Where *Te* represents smooth tensor product function, *Te(air pollutants: meteorological variables)* denotes the interaction term between air pollutants and meteorological variables, and other terms are consistent with DLNMs.

All statistical analyses were performed using SAS (version 9.4, Sai Shi Software, Cary, NC, United States) and R software (version 4.1.2, R Foundation for Statistical Computing, Vienna, Austria). The DLNMs were established using the “dlnm” and “splines” packages.

## 3 Results

### 3.1 Descriptive analysis

Tables 1, 2 present descriptive statistical results for meteorological variables, air pollutants, and EADs due to MBDs during the study period from 2013 to 2020. The average values for daily mean, minimum, and maximum temperature, relative humidity, and wind speed were 23.56°C, 21.15°C, 27.15°C, 75.72%, and 1.94 m/s, respectively. In terms of air pollutants, the mean daily concentrations of PM_2.5_, PM_10_, NO_2_, SO_2_, O_3_, and CO were 28.74 μg/m^3^, 46.87 μg/m^3^, 34.45 μg/m^3^, 8.24 μg/m^3^, 71.40 μg/m^3^, and 0.83 mg/m^3^, respectively. Throughout the study period, a total of 24,967 EADs due to MBDs were recorded, with an average of 8 cases and a maximum of 27 cases per day. Among these cases, approximately 51.2% were male and 43.4% were female. The age groups 15–39 and 40–59 exhibited the highest proportions of EADs due to MBDs, accounting for 62.5% and 24.8% of cases, respectively, while the age groups 0–14 and 60+ accounted for only 1.4% and 5.7%, respectively. Additionally, 1,412 cases did not have recorded age information and 1,360 cases lacked sex information. Due to the small proportion of missing data in this study, no imputation would be performed and these data would not be included in the descriptive analysis.

**Table 1:** Summary of meteorological factors and air pollutants in Shenzhen, 2013–2020.

	Mean ± SD	Min	P_1_	P_10_	P_50_	P_90_	P_99_	Max
*Meteorological factors*								
Tmean, °C	23.56 ± 5.31	3.50	10.20	15.90	24.70	29.60	30.80	33.00
Tmin, °C	21.15 ± 5.41	1.70	7.90	13.40	22.30	27.30	28.60	29.90
Tmax, °C	27.15 ± 5.32	6.50	12.70	19.40	28.20	33.00	35.00	36.90
AT	25.87 ± 7.94	−1.04	6.62	14.42	27.15	34.70	36.42	39.39
RHI	21.53 ± 5.36	2.33	7.30	13.57	23.25	26.98	27.85	29.41
WCI	25.00 ± 6.22	0.61	9.10	16.12	26.37	32.01	33.33	36.10
ET	22.34 ± 4.96	4.46	10.19	15.12	23.25	27.86	28.81	30.07
NET	19.16 ± 6.36	−5.75	2.53	9.90	20.52	26.00	27.29	29.29
Humidex	30.93 ± 9.66	0.69	8.84	16.90	32.17	41.93	44.18	46.79
THIa	24.80 ± 5.75	3.59	10.36	16.46	25.97	31.22	32.26	33.92
Relative humidity, %	75.72 ± 12.88	19.00	34.00	58.00	78.00	90.00	97.00	100.00
Wind speed, m/s	1.94 ± 0.78	0.30	0.80	1.10	1.80	3.00	4.50	6.10
*Daily air pollutants*								
PM_2.5_, μg/m^3^	28.74 ± 17.91	3.13	6.09	10.36	24.91	51.18	91.27	137.07
PM_10_, μg/m^3^	46.87 ± 25.85	5.55	12.91	20.14	40.69	80.35	129.88	181.76
NO_2_, μg/m^3^	34.45 ± 15.93	6.73	11.64	18.09	31.43	54.81	89.26	133.71
SO_2_, μg/m^3^	8.24 ± 3.78	3.09	3.73	4.91	7.36	12.27	23.07	54.81
O_3_, μg/m^3^	71.40 ± 36.36	15.36	21.91	33.96	62.80	122.09	193.00	246.36
CO, mg/m^3^	0.83 ± 0.28	0.40	0.44	0.53	0.76	1.24	1.59	1.93

Tmean, daily mean temperature; Tmin, daily minimum temperature; Tmax, daily maximum temperature; AT, apparent temperature; RHI, Rothfusz’s heat index; WCI, Wind chill index; ET, effective temperature; NET, net effective temperature; Humidex, humidity index; THIa, alternative temperature-humidity index; PM_2.5_, particulate matter less than 2.5 mm in aerodynamic diameter; PM_10_, particulate matter less than 10 mm in aerodynamic diameter; NO_2_, nitrogen dioxide; SO_2_, sulfur dioxide; O_3_, ozone; CO, carbon monoxide; EADs due to MBDs, emergency ambulance dispatches due to mental and behavioral disorders; SD, standard deviation; Min, minimum; Max, maximum; P, percentile.

**Table 2:** Summary of EADs due to MBDs counts in Shenzhen, 2013–2020.

No. of EADs due to MBDs	Sum(%)	Mean ± SD	Min	P_1_	P_10_	P_50_	P_90_	P_99_	Max
Total EADs due to MBDs ^a^	24,967 (100.00)	8.54 ± 4.36	0	1	4	8	14	21	27
*Sex*									
Male	12,784 (51.20)	4.38 ± 2.63	0	0	1	4	8	12	16
Female	10,823 (43.35)	3.70 ± 2.31	0	0	1	3	7	11	16
*Age*									
0–14 years old	359 (1.44)	0.12 ± 0.37	0	0	0	0	1	2	3
15–39 years old	15,592 (62.45)	5.34 ± 2.83	0	0	2	5	9	13	19
40–59 years old	6,182 (24.76)	2.12 ± 1.67	0	0	0	2	4	7	10
60 ~ years old	1,422 (5.70)	0.49 ± 0.74	0	0	0	0	1	3	5

EADs due to MBDs, emergency ambulance dispatches due to mental and behavioral disorders; Sum, total number; SD, standard deviation; Min, minimum; Max, maximum; P, percentile.

a There are 1,360 cases of EADs due to MBDs that lacked gender information and 1,412 cases lacked age information.

Supplementary Figure S2 illustrates the Spearman correlation coefficients between EADs due to MBDs, meteorological variables, and air pollutants. The results showed that the correlation between EADs due to MBDs and meteorological factors was not strong. A strong positive correlation was observed between the daily concentrations of NO_2_ and the daily concentrations of PM_2.5_, PM_10_, SO_2_, and CO (Spearman correlation coefficients *r* > 0.6, *p* < 0.0001). Supplementary Figure S3 presents the time-series distributions of meteorological factors, air pollutants, and daily EADs due to MBDs in Shenzhen from 2013 to 2020. EADs due to MBDs displayed a continuous increase throughout the study period. Additionally, temperature-related indices and relative humidity exhibited distinct annual cycles but no discernible trends. Supplementary Figure S4 shows the monthly characteristics of EADs due to MBDs in Shenzhen from 2013 to 2020. From the heat map, it can be seen that the incidence of mental disorders remained relatively stable in different months throughout the year, and there was no significant fluctuation.

### 3.2 Associations between temperature-related indices and EADs due to MBDs

**Figure 1:**
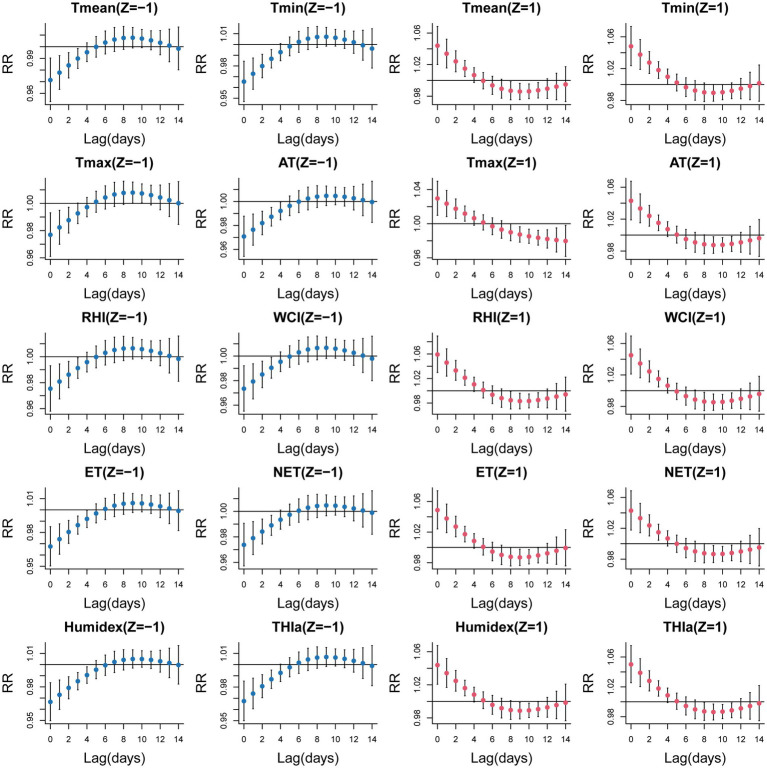
Lag-response curves for ten temperature-related indices associated with EADs due to MBDs at standardized Z values = 1 or −1 (With optimal value as a reference).

**Figure 2:**
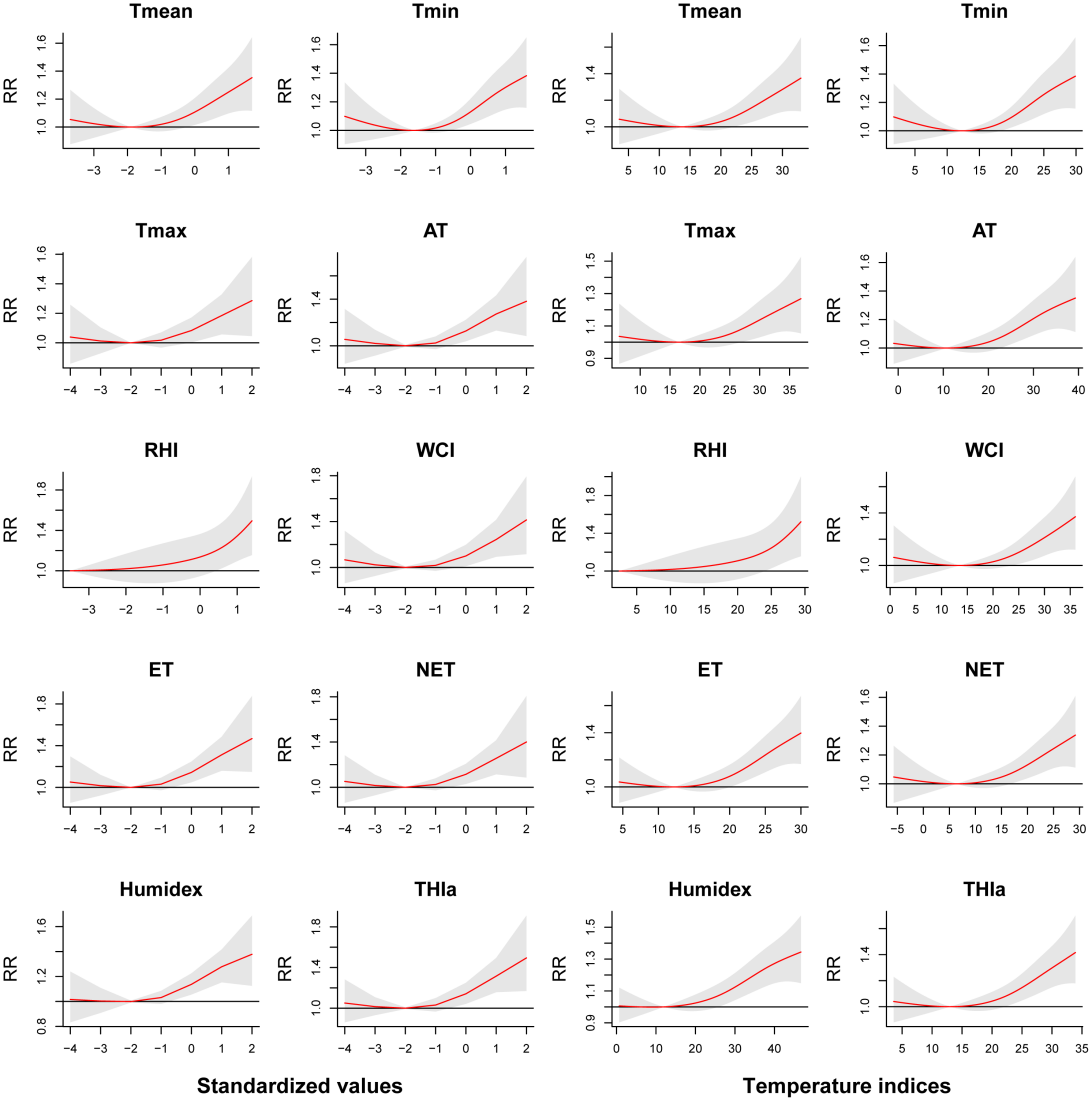
Cumulative relative risks of ten temperature-related indices (standardized values and temperature indices) associated with EADs due to MBDs (lag 0–5).

Figure 1 depicts lag-response curves for 10 temperature-related indices. Using optimal value as the reference for each index, the analysis revealed that the cumulative effects of all indices at *Z* = 1 peaked at lag 0–5. No adverse effects were observed when *Z* = −1. Therefore, we selected lag 0–5 to examine the relationships between temperature-related indices and EADs due to MBDs. Figure 2 shows the cumulative relative risks of 10 temperature-related indices (standardized values and temperature indices) associated with EADs due to MBDs over a 5-day lag period. The optimal values (the point of lowest relative risks) were used as the reference in DLNMs. We revealed a notable influence of high temperature on EADs due to MBDs, whereas low temperature did not demonstrate any statistical significance. Table 3 summarizes the cumulative relative risks of ten temperature-related indices at *Z* = ±1 associated with EADs due to MBDs at lag 0–5. Based on the lowest QAIC value, Humidex was identified as the most suitable temperature-related index linked to EADs due to MBDs. The cumulative relative risk of Humidex at *Z* = 1 was 1.304 (95% CI: 1.158–1.469). Supplementary Table S2 displays the single-day relative risks of temperature-related indices at *Z* = 1 on EADs due to MBDs from 0 to 14 days. The analysis revealed that all 10 temperature-related indices had significant immediate short-term effects on EADs due to MBDs. The relative risk values of all indices peaked on the day of exposure and were mostly detectable up to lag day 3, followed by a declining trend.

**Table 3:** Cumulative relative risks of ten temperature-related indices at Z = ±1 associated with EADs due to MBDs (lag 0–5).

Temperature-related indices	RRs (95% CI) at Z = −1	RRs (95% CI) at Z = 1	QAIC
Tmean	1.019 (0.969–1.072)	**1.250 (1.097–1.423)**	14575.95
Tmin	1.016 (0.984–1.049)	**1.301 (1.146–1.476)**	14571.88
Tmax	1.018 (0.968–1.070)	**1.185 (1.057–1.328)**	14571.74
AT	1.024 (0.971–1.080)	**1.273 (1.131–1.432)**	14569.91
RHI	1.058 (0.881–1.271)	**1.344 (1.096–1.648)**	14571.12
WCI	1.018 (0.971–1.068)	**1.244 (1.093–1.415)**	14574.15
ET	1.031 (0.972–1.093)	**1.312 (1.159–1.486)**	14570.62
NET	1.025 (0.973–1.079)	**1.256 (1.114–1.415)**	14574.82
Humidex	1.028 (0.970–1.089)	**1.304 (1.158–1.469)**	14568.31
THIa	1.031 (0.967–1.099)	**1.315 (1.157–1.494)**	14569.72

EADs due to MBDs, emergency ambulance dispatches due to mental and behavioral disorders; RR, relative risks; CI, confidence intervals; SD, standard deviation; QAIC, Quasi Akaike information criterion; Tmean, daily mean temperature; Tmin, daily minimum temperature; Tmax, daily maximum temperature; AT, apparent temperature; RHI, Rothfusz’s heat index; WCI, wind chill index; ET, effective temperature; NET, net effective temperature; Humidex, humidity index; THIa, alternative temperature-humidity index; With optimal value as a reference; Statistical significance is shown in bold.

**Figure 3:**
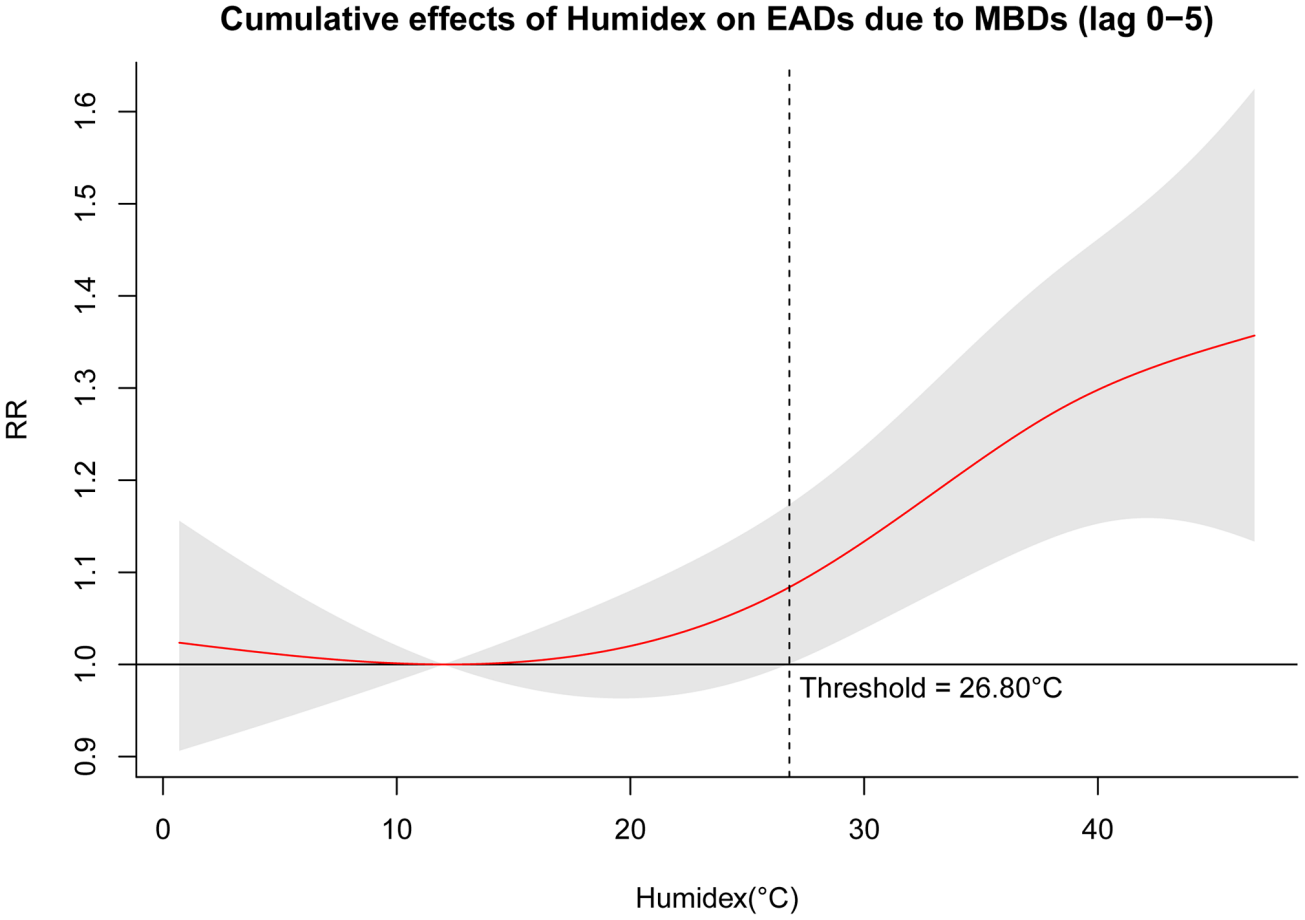
Cumulative relative risks of Humidex associated with EADs due to MBDs at lag 0–5.

Table 4 presents the single-day (lag 0–lag 5) and cumulative (lag 0–1—lag 0–5) effects of Humidex on EADs due to MBDs, at the 1^st^, 10^th^, 90^th^, and 99^th^ percentiles of Humidex compared with Humidex at the lowest risk (3.2^th^ percentile, 12.00°C). Our study found that the 90^th^ percentile (RR = 1.101, 95% CI: 1.056–1.147) and 99^th^ percentile (RR = 1.110, 95% CI: 1.058–1.165) of Humidex indicated a significant short-term effect at lag 0, lasting for 4 days. High temperature (90^th^ percentile, 41.90°C) significantly increased the risk of EADs due to MBDs (RR = 1.318, 95% CI: 1.159–1.499), and a 33.8% increase (RR = 1.338, 95% CI: 1.153–1.553) was observed for exposure to extreme high temperature (99^th^ percentile, 44.20°C) over lag 0–5 days. The cumulative effects between Humidex and EADs due to MBDs over 5 lag days are depicted in Figure 3. The cumulative exposure-response curve between Humidex and EADs due to MBDs exhibited a J-shaped. The result demonstrated that high temperature significantly raised the risk of EADs due to MBDs, while no significant cumulative effects of low temperature was observed at lag 0–5. Additionally, it was observed that the threshold of Humidex for the high temperature effect was 26.80°C (34.6^th^ percentile) at lag 0–5, suggesting that the harmful effect became noticeable when the Humidex exceeded or equaled 26.80°C. Supplementary Figures S5, S6 depict the 3D graph and contour plot, respectively, illustrating the distribution of the effects of different Humidex on EADs due to MBDs across different lag periods. Both figures visually and intuitively illustrated the exposure-lag-response relationship between Humidex and EADs due to MBDs. Simultaneously, for comparison, Supplementary Figure S7 shows the cumulative relative risks of nine other temperature-related indices associated with EADs due to MBDs. It is evident from the figure that all indices exhibit a J-shaped relationship. Similar to Humidex, only high temperatures have an impact on the acute onset of MBDs, although the threshold temperature for each index varies significantly.

**Table 4:** Single-day (lag 0–lag 5) and cumulative (lag 0–1—lag 0–5) effects of Humidex on EADs due to MBDs, at different percentiles of Humidex relative to Humidex at the lowest risk (3.2^th^ percentile, 12.00°C).

Lag days	Relative risk (95% Confidence interval)			
	1^st^ percentile (8.90°C)	10^th^ percentile (16.90°C)	90^th^ percentile (41.90°C)	99^th^ percentile (44.20°C)
Lag0	0.998 (0.988–1.008)	1.006 (0.992–1.019)	**1.101 (1.056–1.147)**	**1.110 (1.058–1.165)**
Lag1	0.999 (0.992–1.007)	1.004 (0.994–1.014)	**1.077 (1.044–1.112)**	**1.084 (1.044–1.125)**
Lag2	1.000 (0.995–1.006)	1.002 (0.994–1.009)	**1.055 (1.030–1.081)**	**1.059 (1.030–1.088)**
Lag3	1.001 (0.997–1.006)	1.000 (0.994–1.006)	**1.035 (1.015–1.054)**	**1.036 (1.013–1.058)**
Lag4	1.002 (0.998–1.006)	0.999 (0.993–1.004)	1.017 (0.999–1.035)	1.016 (0.995–1.037)
Lag5	1.002 (0.997–1.007)	0.998 (0.992–1.004)	1.002 (0.982–1.021)	0.999 (0.977–1.022)
Lag0–1	0.997 (0.980–1.015)	1.009 (0.986–1.032)	**1.186 (1.103–1.276)**	**1.203 (1.105–1.310)**
Lag0–2	0.998 (0.975–1.021)	1.011 (0.981–1.041)	**1.251 (1.137–1.377)**	**1.274 (1.139–1.424)**
Lag0–3	0.999 (0.972–1.026)	1.011 (0.976–1.046)	**1.294 (1.158–1.447)**	**1.319 (1.159–1.501)**
Lag0–4	1.000 (0.972–1.030)	1.009 (0.972–1.047)	**1.316 (1.165–1.486)**	**1.340 (1.164–1.542)**
Lag0–5	1.003 (0.973–1.033)	1.00 (0.968–1.047)	**1.318 (1.159–1.499)**	**1.338 (1.153–1.553)**

EADs due to MBDs, emergency ambulance dispatches due to mental and behavioral disorders; Humidex, humidity index; Statistical significance is shown in bold.

### 3.3 Subgroup analysis

**Figure 4:**
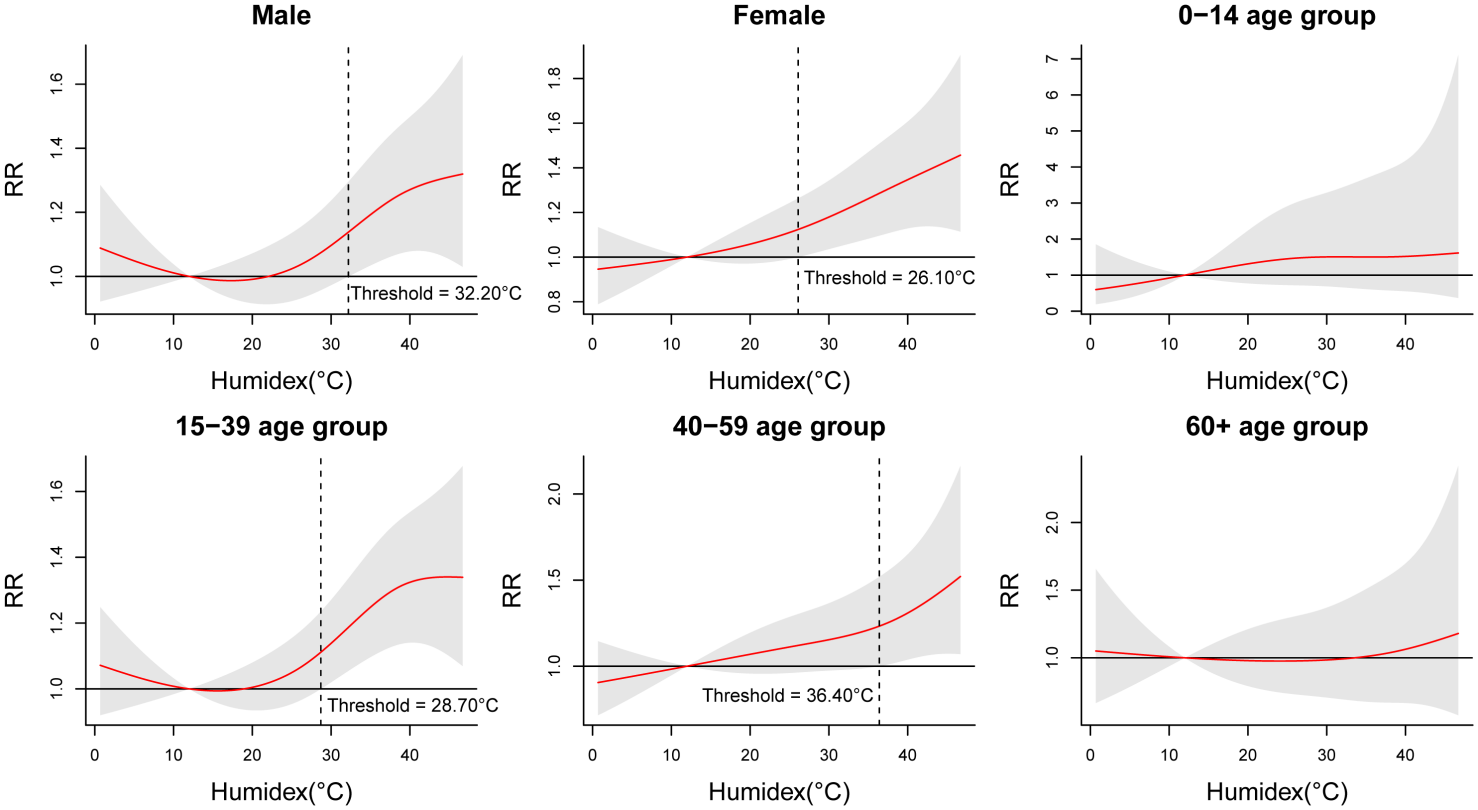
Cumulative relative risks of Humidex associated with EADs due to MBDs stratified by sex and age at lag 0–5.

Table 5 and Figure 4 show the findings of the subgroup analysis. The results indicate a significant association in males, females, 15–39 age group, and 40–59 age group. Among these groups, the highest relative risks were observed for extreme high temperature exposure in the 40–59 age group at lag 0–5 (RR = 1.433, 95% CI: 1.072–1.917). Notably, we did not find significant positive associations in the 0–14 and 60+ age groups. Figure 4 provides the exposure-response curves illustrating the relationship between Humidex and EADs due to MBDs at a lag of 0–5 days. Similarly, only males, females, 15–39 age group, and 40–59 age group displayed consistency with the overall population analysis outcome. The threshold temperatures for these groups were 32.20°C, 26.10°C, 28.70°C, and 36.40°C, respectively. Supplementary Table S3 presents the cumulative effects of Humidex on EADs due to MBDs during the cold and warm seasons. Clearly, the warm season predominantly contributes to this impact, with the highest RR value reaching 1.326 (95% CI: 1.119–1.572) during high Humidex in the warm season.

**Table 5:** Single-day (lag 0–lag 5) and cumulative (lag 0–1—lag 0–5) effects of Humidex on EADs due to MBDs stratified by sex and age, at the 90^th^ and 99^th^ percentiles of Humidex relative to Humidex at the lowest risk.

Lag days	Male	Female	0–14 age group	15–39 age group	40–59 age group	60+ age group
	90^th^ percentile RR (95% CI)					
Lag0	**1.101 (1.040–1.165)**	**1.103 (1.037–1.173)**	1.179 (0.828–1.679)	**1.111 (1.055–1.170)**	**1.088 (1.004–1.179)**	1.033 (0.878–1.216)
Lag1	**1.075 (1.029–1.123)**	**1.082 (1.032–1.135)**	1.135 (0.865–1.489)	**1.084 (1.042–1.128)**	**1.073 (1.008–1.141)**	1.025 (0.904–1.163)
Lag2	**1.051 (1.017–1.086)**	**1.062 (1.025–1.101)**	1.092 (0.892–1.337)	**1.058 (1.027–1.090)**	**1.058 (1.010–1.109)**	1.018 (0.926–1.118)
Lag3	**1.030 (1.003–1.057)**	**1.044 (1.015–1.074)**	1.053 (0.900–1.232)	**1.035 (1.011–1.060)**	**1.044 (1.007–1.084)**	1.010 (0.938–1.089)
Lag4	1.011 (0.986–1.036)	**1.027 (1.000–1.055)**	1.017 (0.880–1.175)	1.014 (0.992–1.037)	1.032 (0.997–1.069)	1.004 (0.936–1.077)
Lag5	0.996 (0.969–1.023)	1.013 (0.984–1.043)	0.984 (0.841–1.152)	0.998 (0.974–1.022)	1.022 (0.984–1.061)	0.998 (0.925–1.077)
Lag0–1	**1.183 (1.070–1.308)**	**1.194 (1.071–1.330)**	1.338 (0.717–2.496)	**1.205 (1.100–1.320)**	**1.167 (1.013–1.346)**	1.060 (0.795–1.413)
Lag0–2	**1.244 (1.090–1.419)**	**1.268 (1.100–1.462)**	1.461 (0.645–3.311)	**1.275 (1.131–1.437)**	**1.235 (1.025–1.489)**	1.078 (0.739–1.574)
Lag0–3	**1.281 (1.099–1.493)**	**1.324 (1.122–1.562)**	1.539 (0.596–3.971)	**1.319 (1.148–1.517)**	**1.290 (1.039–1.603)**	1.090 (0.702–1.691)
Lag0–4	**1.295 (1.095–1.531)**	**1.360 (1.135–1.630)**	1.565 (0.558–4.384)	**1.338 (1.149–1.559)**	**1.332 (1.051–1.688)**	1.094 (0.677–1.768)
Lag0–5	**1.289 (1.079–1.540)**	**1.378 (1.138–1.669)**	1.540 (0.521–4.552)	**1.335 (1.136–1.570)**	**1.361 (1.058–1.749)**	1.092 (0.657–1.816)
	99^th^ percentile RR (95% CI)					
Lag0	**1.112 (1.041–1.188)**	**1.111 (1.034–1.193)**	1.184 (0.789–1.776)	**1.122 (1.057–1.191)**	1.095 (0.997–1.203)	1.044 (0.862–1.265)
Lag1	**1.082 (1.029–1.139)**	**1.089 (1.030–1.150)**	1.139 (0.835–1.555)	**1.090 (1.041–1.142)**	**1.081 (1.005–1.162)**	1.034 (0.893–1.198)
Lag2	**1.055 (1.015–1.096)**	**1.068 (1.025–1.113)**	1.097 (0.871–1.382)	**1.060 (1.024–1.097)**	**1.067 (1.011–1.127)**	1.024 (0.918–1.143)
Lag3	1.030 (0.999–1.061)	**1.048 (1.015–1.083)**	1.057 (0.884–1.264)	**1.033 (1.005–1.062)**	**1.054 (1.010–1.100)**	1.015 (0.932–1.107)
Lag4	1.008 (0.980–1.037)	1.030 (0.999–1.062)	1.020 (0.865–1.203)	1.010 (0.984–1.036)	**1.043 (1.002–1.085)**	1.007 (0.929–1.093)
Lag5	0.991 (0.961–1.022)	1.015 (0.982–1.050)	0.986 (0.823–1.182)	0.991 (0.963–1.019)	1.032 (0.988–1.079)	1.001 (0.915–1.094)
Lag0–1	**1.203 (1.071–1.353)**	**1.209 (1.066–1.372)**	1.349 (0.660–2.757)	**1.223 (1.100–1.359)**	**1.184 (1.003–1.397)**	1.080 (0.770–1.514)
Lag0–2	**1.269 (1.089–1.479)**	**1.291 (1.094–1.524)**	1.479 (0.580–3.775)	**1.296 (1.128–1.490)**	**1.263 (1.016–1.570)**	1.106 (0.710–1.722)
Lag0–3	**1.307 (1.094–1.561)**	**1.353 (1.117–1.640)**	1.564 (0.529–4.625)	**1.339 (1.140–1.574)**	**1.332 (1.035–1.714)**	1.123 (0.672–1.877)
Lag0–4	**1.318 (1.085–1.600)**	**1.394 (1.131–1.719)**	1.595 (0.492–5.175)	**1.352 (1.134–1.613)**	**1.389 (1.055–1.828)**	1.131 (0.647–1.979)
Lag0–5	**1.306 (1.063–1.603)**	**1.416 (1.134–1.767)**	1.573 (0.457–5.419)	**1.340 (1.112–1.615)**	**1.433 (1.072–1.917)**	1.132 (0.627–2.044)

EADs due to MBDs, emergency ambulance dispatches due to mental and behavioral disorders; Humidex, humidity index; RR, relative risks; CI, confidence intervals; Statistical significance is shown in bold.

### 3.4 Sensitivity analysis

Supplementary materials display the results of sensitivity analyses. Supplementary Figure S8 illustrates the temporal variations for two periods (2013–2016 and 2017–2020). Results for both periods exhibit a J-shaped pattern, indicating that only high Humidex values have an impact on EADs due to MBDs. The temporal change did not significantly affect the outcomes. Modifying the degrees of freedom for wind speed and long-term trend did not substantially alter the significant impact of high Humidex on EADs due to MBDs (Supplementary Figures S9, S10). Likewise, adjusting for the maximum lag days did not result in any discernible changes in the cumulative effects (Supplementary Figure S11). After substituting NO_2_ confounding factors with other confounding factors related to pollutants, the outcomes remained relatively unchanged (Supplementary Figure S12). Additionally, after excluding the data post the COVID-19 epidemic, no significant changes were observed in our results (Supplementary Figure S13). The aforementioned sensitivity analyses demonstrate the stability of our results.

### 3.5 Interaction effect analysis

Supplementary Figure S14 depicts the three-dimensional graph illustrating the interaction effect of air pollutants and the Humidex on EADs due to MBDs. It can be observed that for pollutants such as NO_2_, SO_2_, PM_2.5_, and PM_10_, at lower Humidex, an increase in pollutant concentration is associated with a noticeable rise in EADs due to MBDs, reaching its peak at low Humidex and high pollutant concentrations. Conversely, for CO and O_3_, the EADs due to MBDs peaks at high Humidex and relatively higher pollutant concentrations. Our study findings elucidate the interaction effect between pollutants and Humidex on EADs due to MBDs. Specifically, for pollutants like NO_2_, SO_2_, PM_2.5_, and PM_10_, a lower Humidex may enhance the adverse impact of air pollutants on EADs due to MBDs. On the other hand, for CO and O_3_, a higher Humidex may increase their adverse effects on EADs due to MBDs.

## 4 Discussion

This study represents the first investigation to screen various temperature-related indices and assess the influence of the optimal index on EADs due to MBDs in Shenzhen, China. Our findings demonstrated a significant association between exposure to high Humidex and an increase in EADs due to MBDs. The QAIC results indicated that Humidex was the best index for associating with EADs due to MBDs. Additionally, the results showed that high Humidex affected EADs due to MBDs on the day of exposure and lasted for about 4 days. Subgroup analysis results revealed that the effect of high Humidex on EADs due to MBDs was more significant in males, females, 15–39 age group, and 40–59 age group. However, this study did not observe any cold effect on EADs due to MBDs.

Previous studies have primarily focused on examining the association between a single composite index and emergency room visits due to MBDs. A study conducted in Yancheng discovered a connection between increased emergency room admissions for MBDs and short-term exposure to high apparent temperature (23). In recent years, other comprehensive indices have become widely used in meteorological research. For instance, a study in Romania assessed summer thermal comfort using the net effective temperature index (39). Carder et al. found that the wind chill index is considered to be a better index than other temperature-related indices for estimating the impact of cold on health (40). However, no studies have examined comprehensive indices to determine the best index for exploring the influence of temperature on EADs due to MBDs. Consequently, there is currently no temperature index and threshold suitable for subtropical areas to protect the mental health of individuals. By utilizing a standardized method that eliminates the influence of dimensions, our study allows for direct comparisons between different temperature-related indices. Humidex, proposed by Masterton and Richardson, is a temperature-humidity combination index utilized to reflect human-perceived heat, widely employed by Canadian meteorologists to describe an individual’s thermal comfort in hot or humid weather (27). Compared with a single index, Humidex more comprehensively captures the combined impact of temperature and humidity on mental health. Compared with other comprehensive indices, it is a thermal comfort index that specifically describes hot and humid weather. Similarly, Humidex was also selected as the research index in the previous study on temperature and depression (41).

Recent studies have shown a significant association between high ambient temperatures and hospital admissions for MBDs (17, 42, 43). In Shanghai, the cumulative relative risk of high temperature (33.10°C) was 1.266 (95% CI: 1.074–1.493) at lag 0–1 (14). A study in Ho Chi Minh City demonstrated a strong correlation between the main and added effects of heat waves on MBDs (42). However, conflicting conclusions were drawn in other studies. A case-crossover study in Beijing indicated that both low and high apparent temperatures could contribute significantly to emergency visits for psychiatric disorders (15). This discrepancy may be attributed to the higher latitude and lower average temperature in Beijing compared to Shenzhen, where the effect of low temperature is more noticeable. A previous study in Shenzhen investigated the impact of ambient temperature on emergency ambulance dispatches due to MBDs, reporting relative risk values of 1.01 (95% CI: 0.61–1.70) for the heat effect and 1.26 (95% CI: 1.06–1.51) for the cold effect (44). However, this study had limitations, including a small sample size resulting from data collection only from 2015 to 2016, as well as stratification by season, which hindered the observation of existing effects. In our study, we observed J-shaped cumulative exposure–response curves, where EADs due to MBDs increased at high Humidex. Harvesting effects of high Humidex were noted in our study, leading to an overestimation of EADs due to MBDs attributable to high Humidex. Several factors were suggested to potentially impact the appearance of these harvesting effects, including the socioeconomic and baseline health status of the population, interactions between air pollutants and meteorological variables, and the choice of model parameters. High Humidex was found to hasten the onset of mental disorders in individuals already in a high-risk pool but did not cause the recruitment of new individuals into the pool, resulting in the observed harvesting effects. Shenzhen, being a relatively young economically developed city with a constant influx of population, particularly in the younger age groups, may experience an increase in the incidence of high-risk individuals due to high Humidex. However, the rate of recruitment of new individuals into the high-risk group may not increase at the same rate.

Age and sex play a crucial role in influencing the occurrence of EADs due to MBDs associated with high temperatures. Previous studies conducted in South Korea and California have suggested that the older adult constitute a susceptible population (29, 43). However, conflicting results from other studies have proposed that young individuals or those under 45 years old may be more susceptible to high temperature compared to the older adult (13, 23). Variations in the results of age subgroups may stem from differences in the demographic composition of various cities and the neglect of individual socio-economic factors. Notably, Shenzhen is an immigrant city with a sizable young population, potentially making young people more prone to mental disorders acute attacks triggered by high temperature. Regarding gender differences, some studies have suggested that women are more susceptible (14, 17), while others have indicated that men are at higher risk (15, 42). Discrepancies in these findings could be attributed to physiological variations (45), lifestyle differences (46, 47), and adaptability between genders (48, 49). Therefore, further research is necessary to enhance our understanding of the effect of gender on the relationship between temperature and mental disorders.

Furthermore, different geographical areas exhibit varying threshold temperatures for the heat effect. Our study has identified a Humidex threshold for the high temperature effect at 26.80°C. The Humidex thresholds for males, females, 15–39 age group, and 40–59 age group are 32.20°C, 26.10°C, 28.70°C, and 36.40°C, respectively. It is important to note that the ambient temperature threshold in Shanghai is reported to be 24.60°C (14), while in northern Vietnam it can be as high as 35.00°C (42). These discrepancies in results may be attributed to different statistical models, temperature indices, study locations, or demographic factors. In addition, our study also found there may be interaction between pollutants and meteorological factors, and more accurate models are needed to quantitatively analyze the impact of interaction on mental disorders in the future.

The biological mechanism underlying the associations between meteorological variables and MBDs has been widely reported. MBDs encompass disorders of brain functional activities, influencing mental aspects such as cognition, emotion, behavior, and will to varying degrees. Patients with MBDs have diverse genetic backgrounds and living environments. However, the specific mechanism by which meteorological factors increase the incidence of MBDs remains unclear. On one hand, MBDs patients often experience cognitive impairments that hinder their ability to understand and respond to extreme weather conditions. This lack of appropriate evasive measures further contributes to the development of these disorders (50). Additionally, certain psychotropic drugs may interfere with the body’s temperature regulation system, increasing susceptibility to temperature changes (51). Neurotransmitters such as dopamine, serotonin, and norepinephrine are known to regulate body temperature through the hypothalamus-pituitary-thyroid and hypothalamus-pituitary–adrenal axes (52–54). Consequently, high temperature has been found to negatively impact cognitive function, leading to increased plasma serotonin levels and dopamine suppression (55). In the context of schizophrenia, the dopamine hypothesis suggests that an excess of dopamine activity may underlie its symptoms (56). Similarly, patients with bipolar disorder commonly exhibit symptoms such as mood swings, anxiety, and insomnia, which are associated with abnormal dopamine and serotonin levels (57).

This study builds upon previous literature that explored the influence of temperature on MBDs. Our research has several strengths. Firstly, EADs data may be more responsive to temperature and humidity changes compared to hospitalization data. The EADs were collected from the Shenzhen First-aid Command Center which handles calls from the entire city and covers a significant portion of the permanent population in Shenzhen. Therefore, the results obtained in this study may accurately represent the relationship between temperature-related indices and EADs due to MBDs. Secondly, we standardized and screened ten different temperature-related indices in our study. This approach allowed us to compare these indices and identify the most suitable one for investigating the relationship between temperature and EADs due to MBDs through standardization and QAIC. Thirdly, after selecting the optimal index, we specifically studied the immediate and lagged effect of Humidex on EADs due to MBDs at different percentiles and identified the threshold temperature. However, there are some limitations that should be addressed. Firstly, the EADs data did not include detailed individual medical records, which may have resulted in inappropriate MBDs diagnoses. Detailed diagnostic data are needed in the future to enhance the reliability of the obtained MBDs patient data. Secondly, the local weather data were based on station monitoring data rather than direct measurements of individuals’ real environmental exposure. This might have contributed to exposure misclassification to some extent in our study. Additionally, we lacked information on personal lifestyle, socioeconomic status, occupation, education level, etc. Consequently, we were unable to adjust for the impact of these factors on our models and results. Future research should consider incorporating these variables into the models to enhance the accuracy of the results.

## 5 Conclusion

Our study indicates that Humidex was the most appropriate index for evaluating the short-term effects of temperature on EADs due to MBDs. In subtropical areas like Shenzhen, high Humidex may increase the risk of EADs caused by MBDs. When Humidex exceeds 26.80°C, the high temperature effect gradually becomes significant. With the ever-growing impact of global warming, the number of MBDs linked to high temperatures may escalate, exacerbating the disease burden. Based on this study, policymakers in the healthcare sector could enhance relevant legislation and regulations based on measurement indices, critical temperature thresholds, and vulnerable populations. Additionally, there should be a reasonable allocation of medical resources to accommodate the potential surge in emergency treatments. Social institutions specific to this issue can notify the public about anticipated hot and humid weather conditions and their duration, providing detailed information on the adverse effects of high temperature on the human body and opening public cooling centers. Individuals, especially susceptible people should receive high temperature warning information in advance and take cooling measures such as using air conditioners or drinking cold beverages, thus reducing the number of mental disorders requiring emergency treatment in high Humidex environments.

## Data availability statement

The raw data supporting the conclusions of this article will be made available by the authors, without undue reservation.

## Author contributions

ZiY: Conceptualization, Methodology, Software, Data curation, Writing–original draft, Writing–review & editing. MJ: Data curation, Writing–review & editing. ZhY: Writing–review & editing. SC: Writing–review & editing. SH: Resources, Supervision, Writing–review & editing. JC: Resources, Supervision, Writing–review & editing. XL: Resources, Supervision, Writing–review & editing. NL: Resources, Supervision, Writing–review & editing. PW: Data curation, Writing–review & editing. PY: Supervision, Project administration, Funding acquisition, Writing–review & editing. HJ: Methodology, Writing–review & editing, Supervision.
