# Supplementary material for: Short-term effects of temperature-related indices on emergency ambulance dispatches due to mental and behavioral disorders in Shenzhen, China
Source: Front Public Health. 2024 May 30;12:1343550. doi: 10.3389/fpubh.2024.1343550 (PMC11177611; doi:10.3389/fpubh.2024.1343550)
Supplement: Supplementary file 1 [file Data_Sheet_1.docx]

Supplementary Material

# Supplementary Tables

**Table S1.** Summary of temperature-related indices used in this study.

| Abbreviation | Names of indices | Definition/equation |
| --- | --- | --- |
| Raw temperature-related indices | | |
| Tmean | daily mean temperature | Daily 24-hour average |
| Tmin | daily minimum temperature | The minimum hourly temperature in a day |
| Tmax | daily maximum temperature | The maximum hourly temperature in a day |
| Composite temperature-related indices | | |
| AT | apparent temperature | $AT=T\text{mean}+0.33(\frac{RH}{100}*6.105*\exp(\frac{17.27Tmean}{237.7+Tmean}))-0.70WS-4.00$ |
| RHI | Rothfusz's heat index |  |
| WCI | wind chill index |  |
| ET | effective temperature |  |
| NET | net effective temperature |  |
| Humidex | humidity index |  |
| THIa | alternative temperature-humidity index |  |

Abbreviations: Tmean, daily mean temperature(℃); Tmin, daily minimum temperature(℃); Tmax, daily maximum temperature(℃); RH, relative humidity(%); WS, wind speed(m/s); c_i_ in the equation of Rothfusz's heat index are all constants and the corresponding values are as follows:$\text{c}_{\text{1}}$ = -8.78469475556,$\text{c}_{\text{2}}$ = 1.61139411,$\text{c}_{\text{3}}$ = 2.33854883889,$\text{c}_{\text{4}}$ = -0.14611605,$\text{c}_{\text{5}}$ = -0.012308094,$\text{c}_{\text{6}}$ = -0.0164248277778,$\text{c}_{\text{7}}$ = 0.002211732, $\text{c}_{\text{8}}$= 0.00072546,$\text{c}_{\text{9}}$ = -0.000003582.

**Table S2.** Single-day relative risks of temperature-related indices at Z = 1 associated with EADs due to MBDs.

| Lag days | Tmean | Tmin | Tmax | AT | RHI | WCI | ET | NET | Humidex | THIa |
| --- | --- | --- | --- | --- | --- | --- | --- | --- | --- | --- |
| Lag0 | **1.044(1.020-1.068)** | **1.048(1.024-1.073)** | **1.030(1.010-1.050)** | **1.043(1.019-1.067)** | **1.059(1.030-1.089)** | **1.045(1.021-1.070)** | **1.049(1.024-1.074)** | **1.043(1.018-1.069)** | **1.044(1.021-1.067)** | **1.050(1.025-1.075)** |
| Lag1 | **1.034(1.016-1.052)** | **1.038(1.019-1.057)** | **1.024(1.008-1.039)** | **1.033(1.015-1.052)** | **1.046(1.024-1.068)** | **1.035(1.016-1.053)** | **1.038(1.019-1.057)** | **1.033(1.014-1.052)** | **1.034(1.017-1.052)** | **1.039(1.020-1.058)** |
| Lag2 | **1.024(1.011-1.038)** | **1.028(1.014-1.041)** | **1.018(1.006-1.029)** | **1.024(1.011-1.037)** | **1.033(1.017-1.049)** | **1.024(1.011-1.038)** | **1.027(1.014-1.041)** | **1.024(1.010-1.037)** | **1.025(1.012-1.037)** | **1.028(1.015-1.042)** |
| Lag3 | **1.015(1.005-1.025)** | **1.018(1.008-1.029)** | **1.012(1.003-1.021)** | **1.015(1.006-1.025)** | **1.021(1.009-1.034)** | **1.015(1.005-1.025)** | **1.017(1.007-1.028)** | **1.015(1.005-1.025)** | **1.016(1.007-1.026)** | **1.018(1.008-1.028)** |
| Lag4 | 1.007(0.997-1.016) | **1.010(1.000-1.019)** | 1.006(0.998-1.015) | 1.007(0.998-1.017) | 1.010(0.999-1.022) | 1.006(0.997-1.016) | 1.008(0.999-1.018) | 1.007(0.997-1.017) | 1.008(0.999-1.017) | 1.009(0.999-1.018) |
| Lag5 | 1.000(0.989-1.010) | 1.002(0.992-1.013) | 1.001(0.993-1.010) | 1.001(0.990-1.011) | 1.001(0.988-1.014) | 0.999(0.989-1.009) | 1.001(0.990-1.012) | 1.000(0.989-1.011) | 1.001(0.991-1.011) | 1.001(0.990-1.012) |
| Lag6 | 0.994(0.982-1.005) | 0.997(0.985-1.008) | 0.997(0.987-1.007) | 0.995(0.983-1.007) | 0.994(0.979-1.008) | 0.993(0.982-1.005) | 0.995(0.982-1.007) | 0.994(0.982-1.007) | 0.996(0.984-1.007) | 0.994(0.982-1.007) |
| Lag7 | 0.989(0.977-1.002) | 0.992(0.980-1.005) | 0.993(0.983-1.004) | 0.991(0.978-1.003) | 0.988(0.973-1.003) | 0.989(0.977-1.001) | 0.990(0.978-1.003) | 0.990(0.977-1.003) | 0.992(0.980-1.004) | 0.990(0.977-1.003) |
| Lag8 | 0.987(0.975-0.998) | 0.990(0.978-1.002) | 0.990(0.980-1.000) | 0.988(0.977-1.000) | 0.985(0.970-0.999) | 0.986(0.975-0.998) | 0.988(0.976-1.000) | 0.988(0.975-1.000) | 0.989(0.978-1.001) | 0.987(0.975-0.999) |
| Lag9 | 0.986(0.976-0.996) | 0.990(0.979-1.000) | 0.987(0.978-0.996) | 0.987(0.977-0.998) | 0.983(0.971-0.996) | 0.985(0.975-0.996) | 0.987(0.976-0.998) | 0.987(0.976-0.998) | 0.989(0.979-0.999) | 0.986(0.976-0.997) |
| Lag10 | 0.986(0.977-0.996) | 0.990(0.981-1.000) | 0.985(0.977-0.994) | 0.988(0.979-0.997) | 0.983(0.972-0.995) | 0.986(0.976-0.995) | 0.988(0.978-0.997) | 0.987(0.977-0.997) | 0.989(0.980-0.998) | 0.987(0.977-0.996) |
| Lag11 | 0.987(0.978-0.997) | 0.992(0.982-1.002) | 0.984(0.975-0.992) | 0.989(0.980-0.998) | 0.985(0.973-0.997) | 0.987(0.978-0.997) | 0.990(0.980-0.999) | 0.988(0.978-0.998) | 0.991(0.982-1.000) | 0.989(0.979-0.999) |
| Lag12 | 0.990(0.977-1.002) | 0.995(0.982-1.008) | 0.982(0.971-0.993) | 0.991(0.978-1.003) | 0.987(0.972-1.003) | 0.990(0.977-1.002) | 0.992(0.979-1.005) | 0.990(0.977-1.003) | 0.993(0.981-1.005) | 0.991(0.978-1.004) |
| Lag13 | 0.992(0.975-1.009) | 0.998(0.981-1.016) | 0.981(0.967-0.995) | 0.993(0.976-1.011) | 0.991(0.970-1.012) | 0.993(0.976-1.010) | 0.996(0.978-1.014) | 0.992(0.974-1.011) | 0.995(0.979-1.012) | 0.994(0.977-1.012) |
| Lag14 | 0.995(0.973-1.017) | 1.002(0.979-1.025) | 0.980(0.962-0.998) | 0.996(0.973-1.019) | 0.994(0.967-1.022) | 0.996(0.974-1.018) | 0.999(0.976-1.023) | 0.995(0.971-1.020) | 0.998(0.977-1.021) | 0.998(0.975-1.022) |

Abbreviations: Tmean, daily mean temperature; Tmin, daily minimum temperature; Tmax, daily maximum temperature; AT, apparent temperature; RHI, Rothfusz's heat index; WCI, wind chill index; ET, effective temperature; NET, net effective temperature; Humidex, humidity index; THIa, alternative temperature-humidity index; With optimal value as a reference.

**Table S3.** The relative risks of Humidex associated with EADs due to MBDs stratified by season for different levels of Humidex.

| Lag days | Cold season | | Warm season | |
| --- | --- | --- | --- | --- |
|  | 1st percentile (8.90°C) | 10th percentile (16.90°C) | 90th percentile (41.90°C) | 99th percentile (44.20°C) |
| Lag0 | 1.011(0.976-1.048) | 1.000(0.998-1.003) | **1.123(1.053-1.199)** | **1.128(1.048-1.214)** |
| Lag0–1 | 1.020(0.957-1.086) | 1.001(0.996-1.005) | **1.123(1.091-1.371)** | **1.231(1.081-1.401)** |
| Lag0–3 | 1.028(0.932-1.135) | 1.001(0.994-1.008) | **1.326(1.119-1.572)** | **1.331(1.093-1.620)** |
| Lag0–5 | 1.028(0.914-1.155) | 1.001(0.993-1.009) | **1.299(1.074-1.572)** | **1.290(1.029-1.618)** |

Abbreviations: Warm season from April 20th to November 7th, and the rest (from November 8th to April 19th of the following year) is grouped as cold season; Statistically significant results (*p*<0.05) are marked in bold; With optimal value as a reference.

# Supplementary Figures


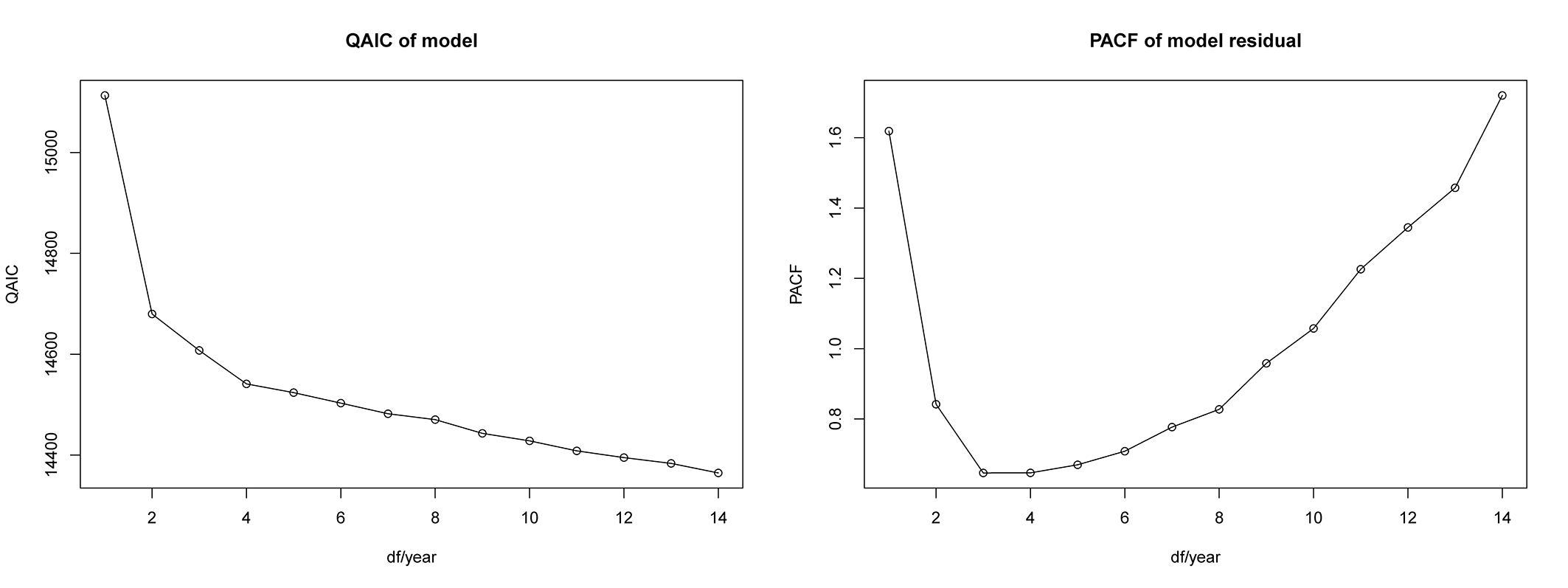


**Fig. S1.** Choosing the degrees of freedom of long-term trend based on QAIC and PACF.

Abbreviations: QAIC, Quasi Akaike information criterion; PACF, Partial autocorrelation coefficient.

**
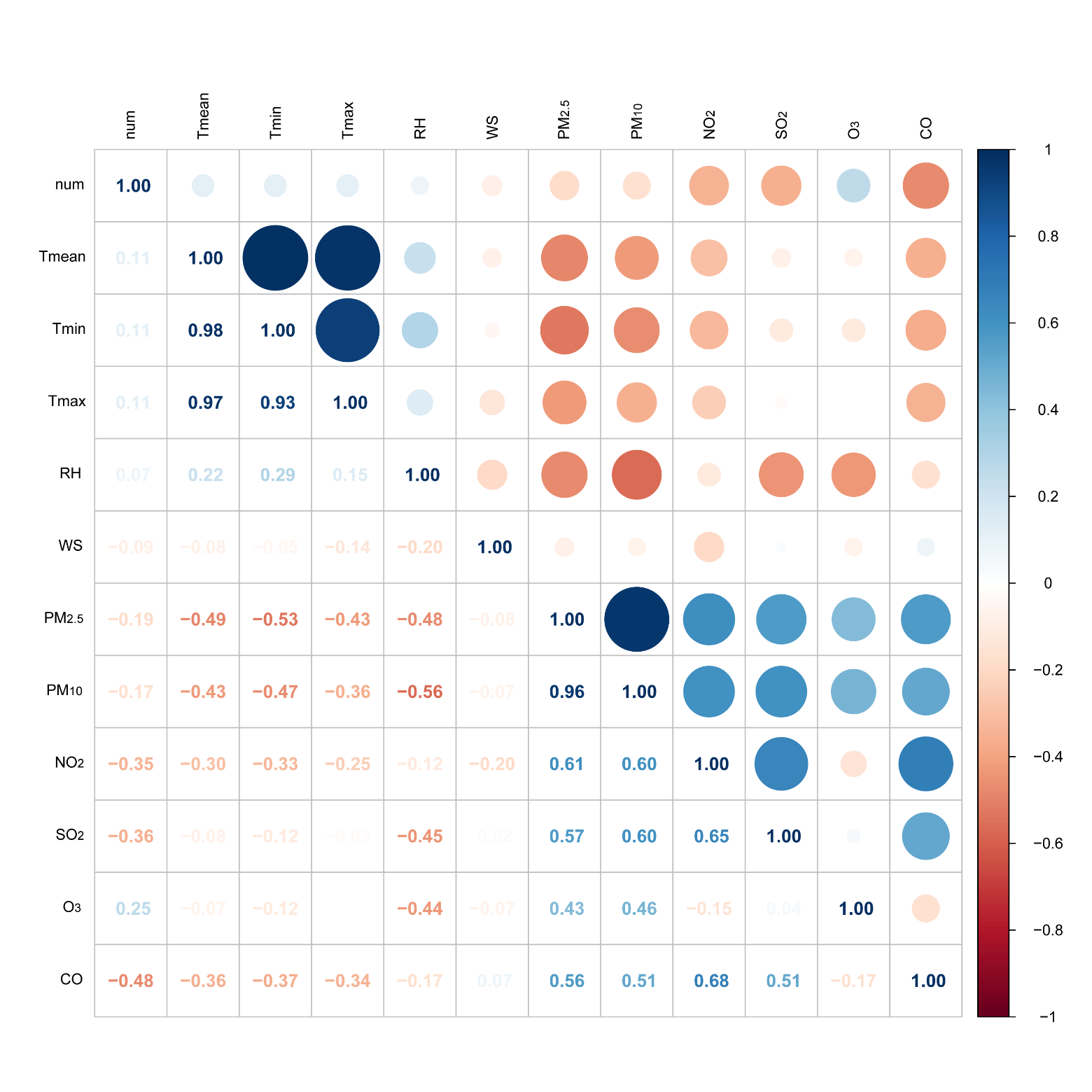
**

**Fig. S2.** Spearman’s correlation coefficients between EADs due to MBDs, meteorological factors, and air pollutants in Shenzhen, 2013–2020.

Abbreviations: Tmean, daily mean temperature; Tmin, daily minimum temperature; Tmax, daily maximum temperature; RH, relative humidity; WS, wind speed; PM_2.5_, particulate matter less than 2.5mm in aerodynamic diameter; PM_10_, particulate matter less than 10mm in aerodynamic diameter; NO_2_, nitrogen dioxide; SO_2_, sulfur dioxide; O_3_, ozone; CO, carbon monoxide.


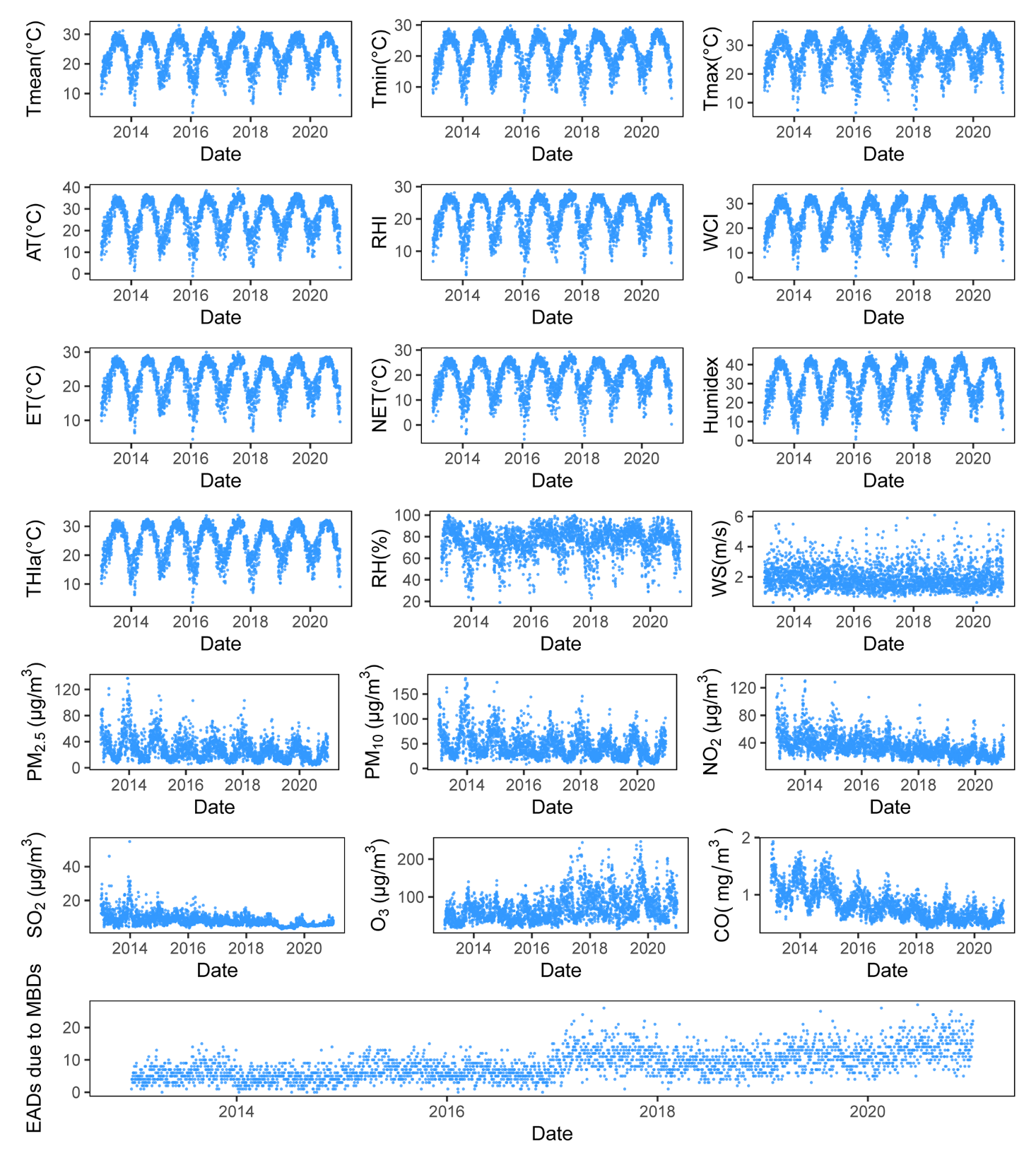


**Fig. S3.** Time-series plots for daily meteorological factors, air pollutants, and EADs due to MBDs in Shenzhen, 2013–2020.

Abbreviations: Tmean, daily mean temperature; Tmin, daily minimum temperature; Tmax, daily maximum temperature; AT, apparent temperature; RHI, Rothfusz's heat index; WCI, wind chill index; ET, effective temperature; NET, net effective temperature; Humidex, humidity index; THIa, alternative temperature-humidity index; RH, relative humidity; WS, wind speed; PM_2.5_, particulate matter less than 2.5mm in aerodynamic diameter; PM_10_, particulate matter less than 10mm in aerodynamic diameter; NO_2_, nitrogen dioxide; SO_2_, sulfur dioxide; O_3_, ozone; CO, carbon monoxide; EADs due to MBDs, emergency ambulance dispatches due to mental and behavioral disorders.


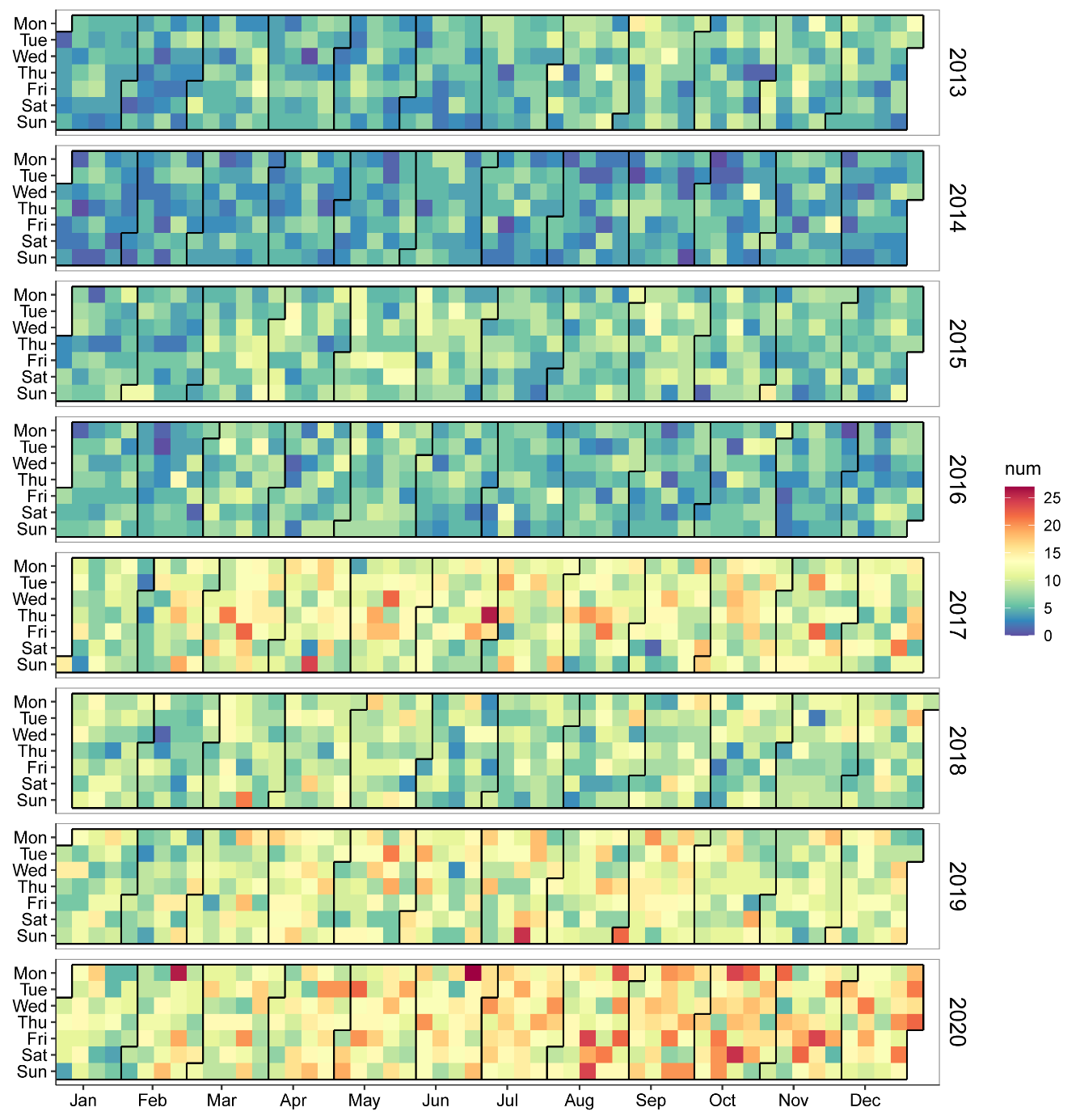


**Fig. S4.** Monthly characteristics of EADs due to MBDs in Shenzhen, 2013–2020.


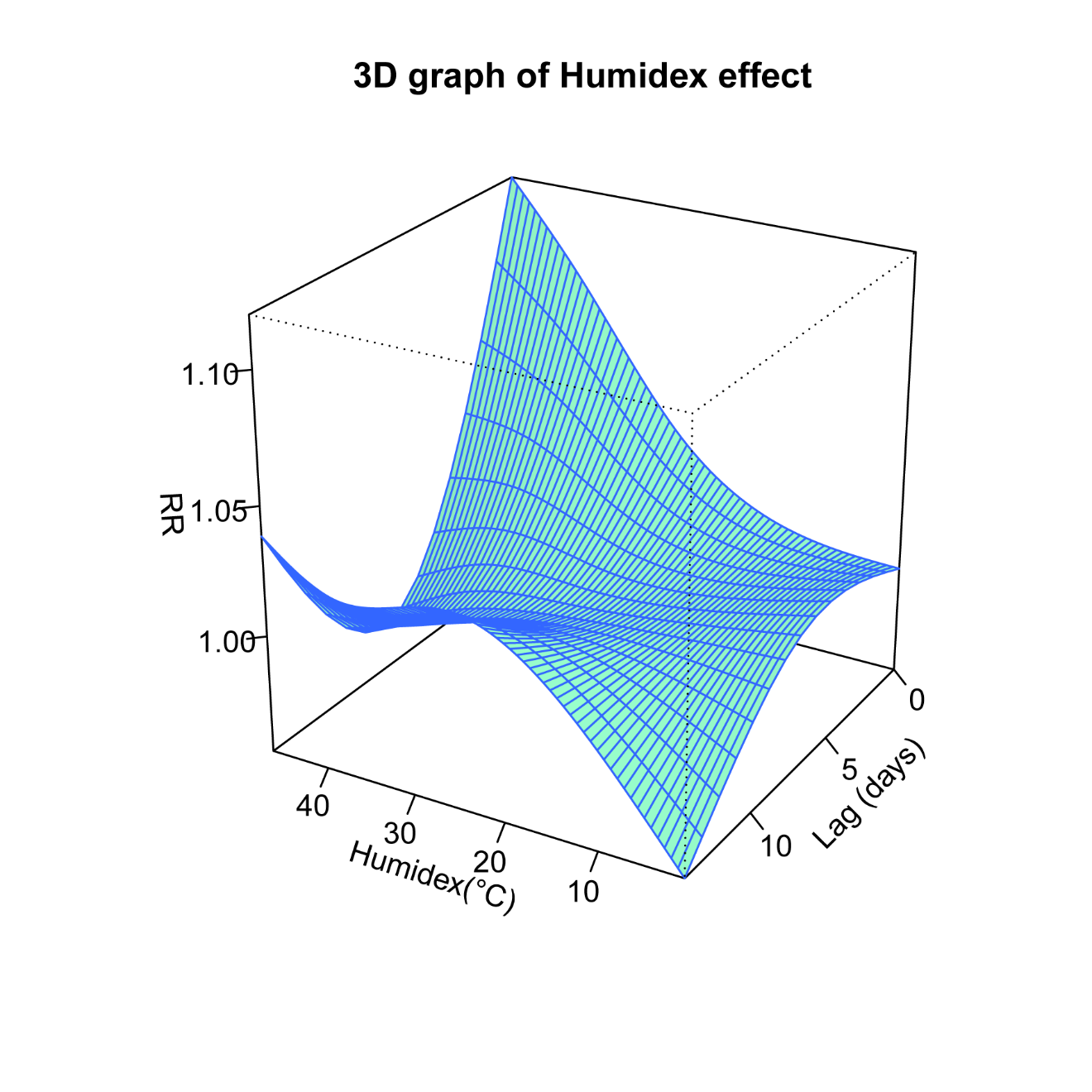


**Fig. S5.** 3D graph illustrating the distribution of the effects of different Humidex on EADs due to MBDs across different lag periods.

Abbreviations: EADs due to MBDs, emergency ambulance dispatches due to mental and behavioral disorders; Humidex, humidity index.


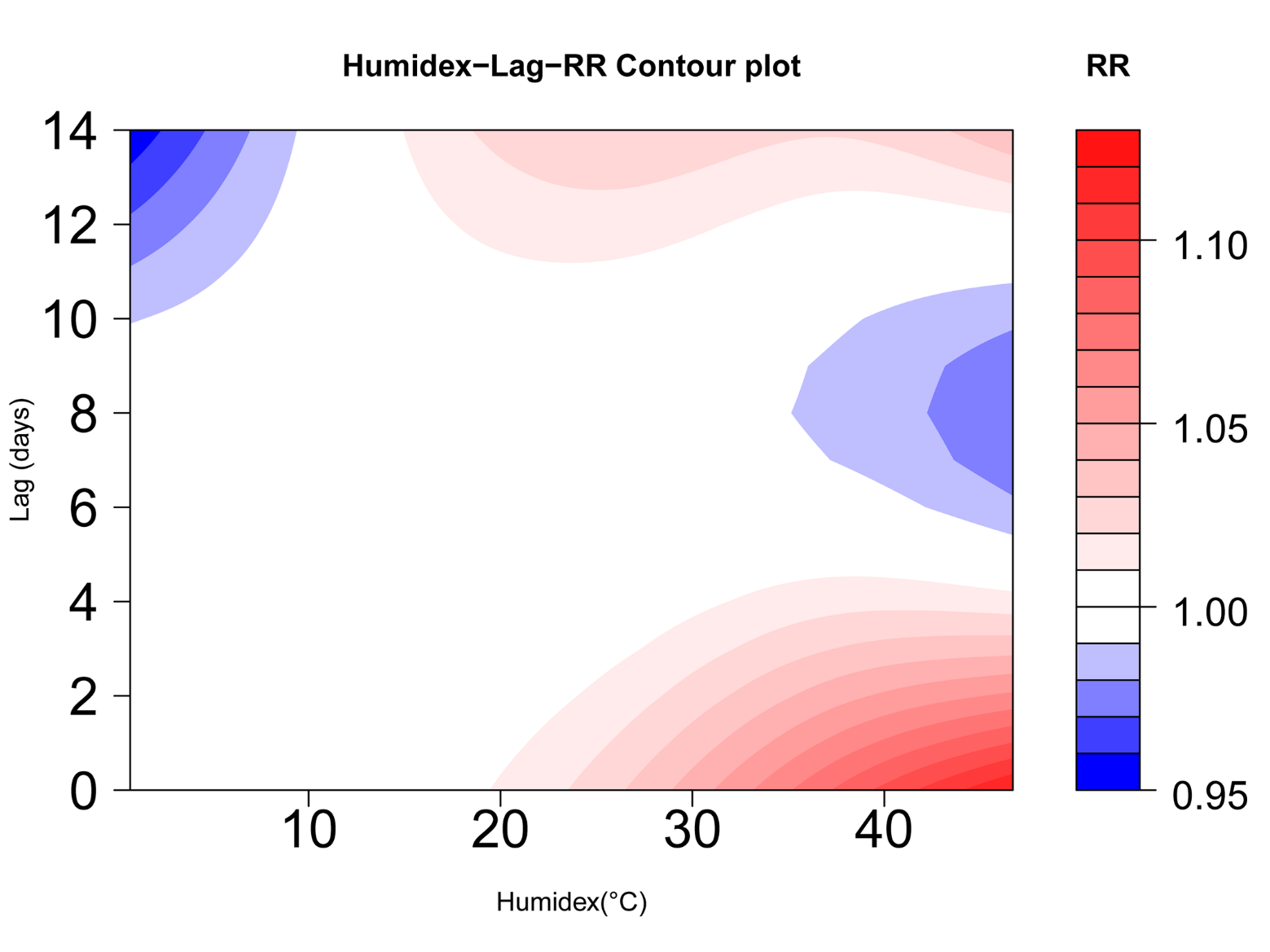


**Fig. S6.** Contour plot depicting the effects of different Humidex on EADs due to MBDs across different lag periods.

Abbreviations: EADs due to MBDs, emergency ambulance dispatches due to mental and behavioral disorders; Humidex, humidity index.


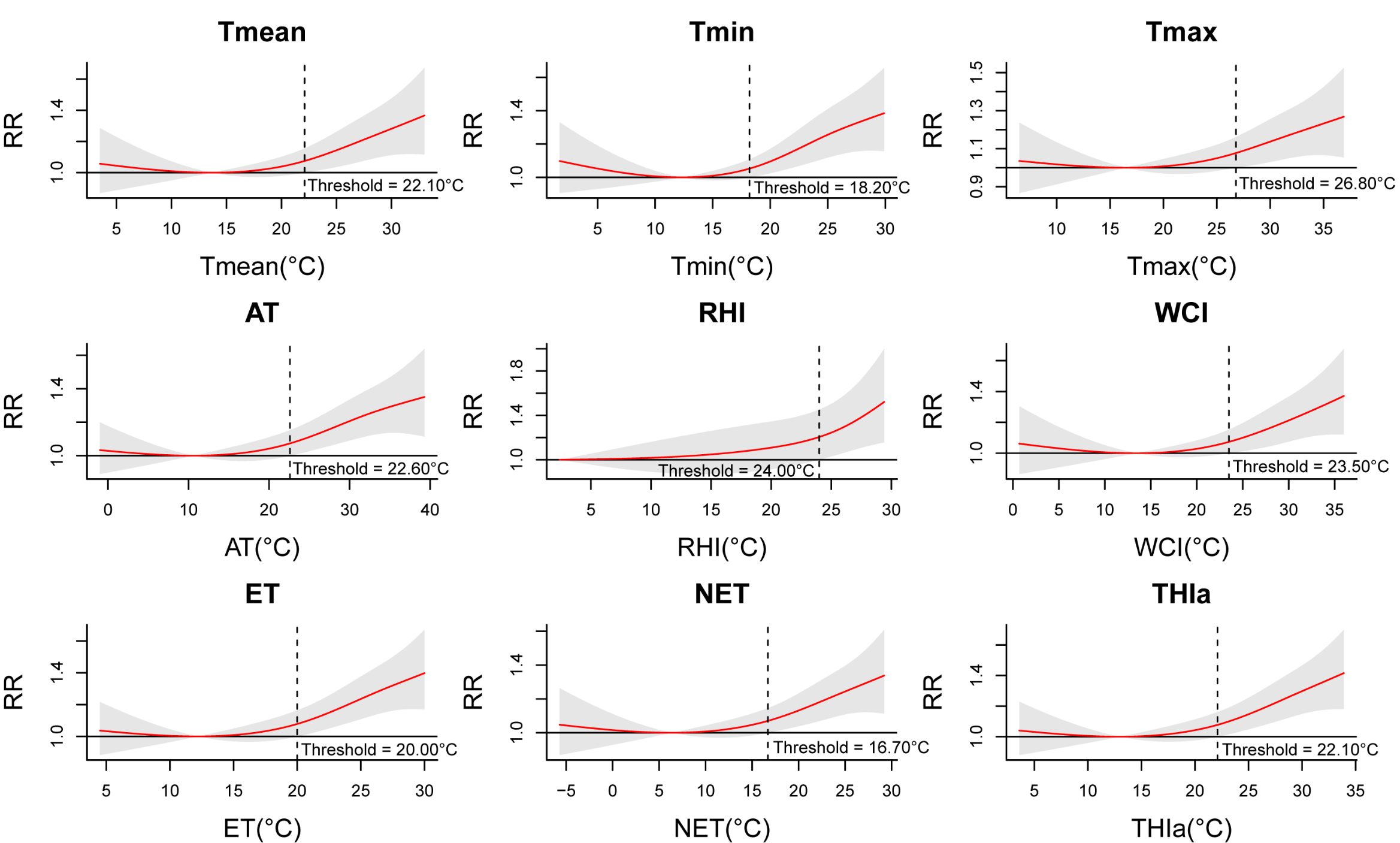


**Fig. S7.** Cumulative relative risks of temperature-related indices associated with EADs due to MBDs at lag 0–5.

Abbreviations: EADs due to MBDs, emergency ambulance dispatches due to mental and behavioral disorders; Tmean, daily mean temperature; Tmin, daily minimum temperature; Tmax, daily maximum temperature; AT, apparent temperature; RHI, Rothfusz's heat index; WCI, wind chill index; ET, effective temperature; NET, net effective temperature; THIa, alternative temperature-humidity index; With optimal value as a reference.


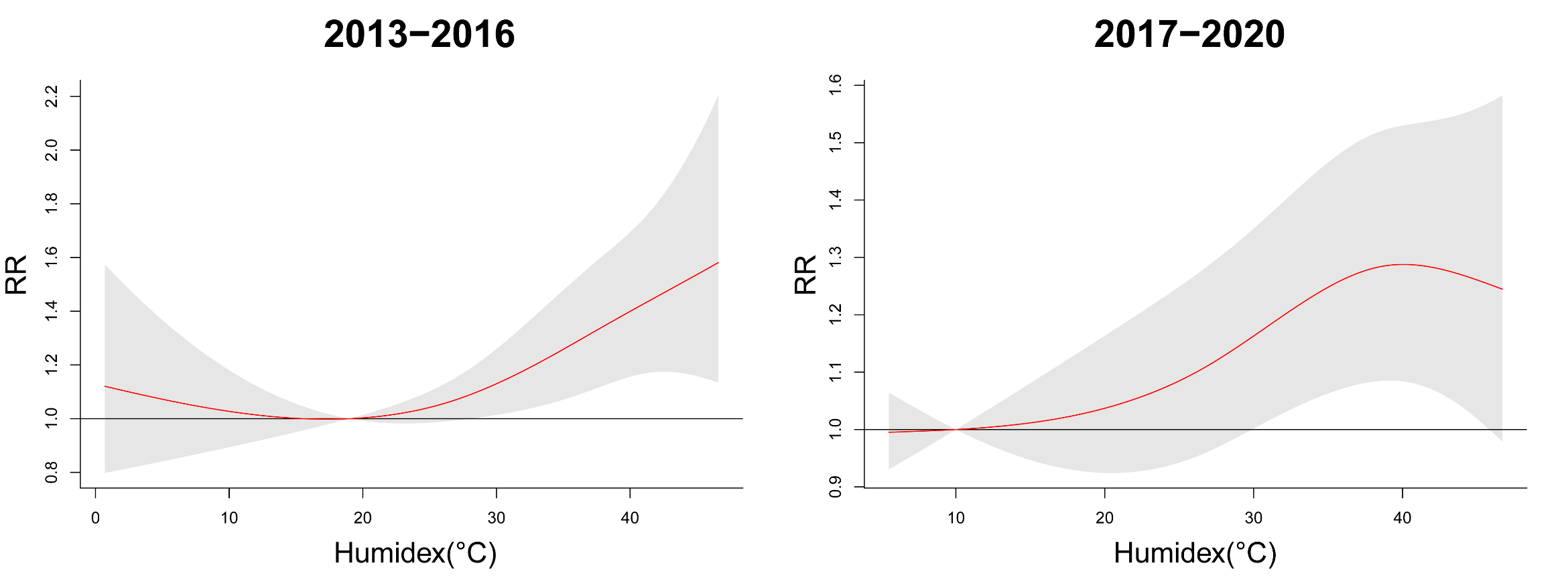


**Fig. S8.** Sensitivity analysis illustrating the temporal variations associated with two periods (2013-2016 and 2017-2020)

Abbreviations: Humidex, humidity index.


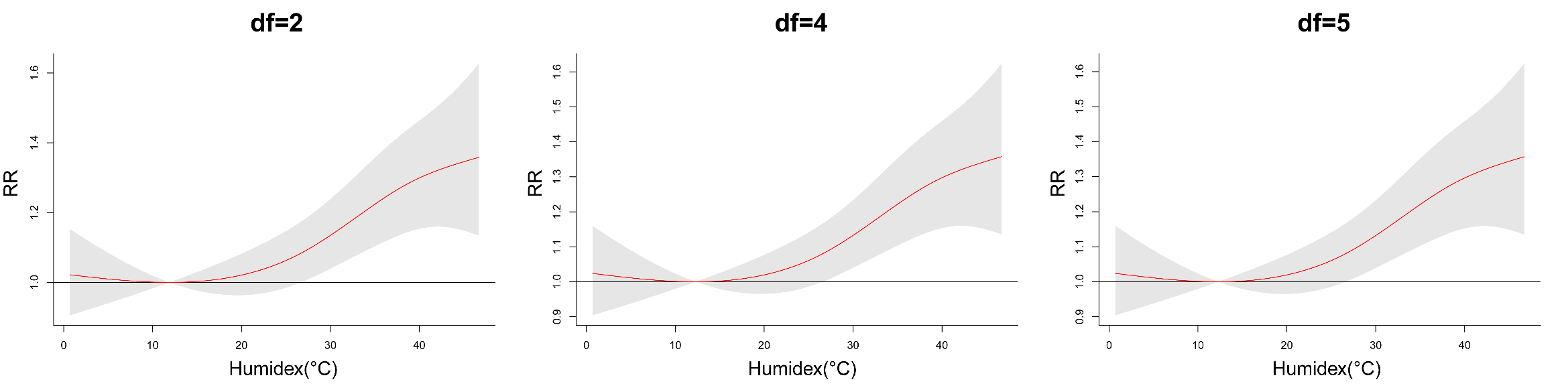


**Fig. S9.** Sensitivity analysis when altering the degrees of freedom (*df* = 2, 4, 5) for wind speed (lag 0–5).

Abbreviations: Humidex, humidity index.


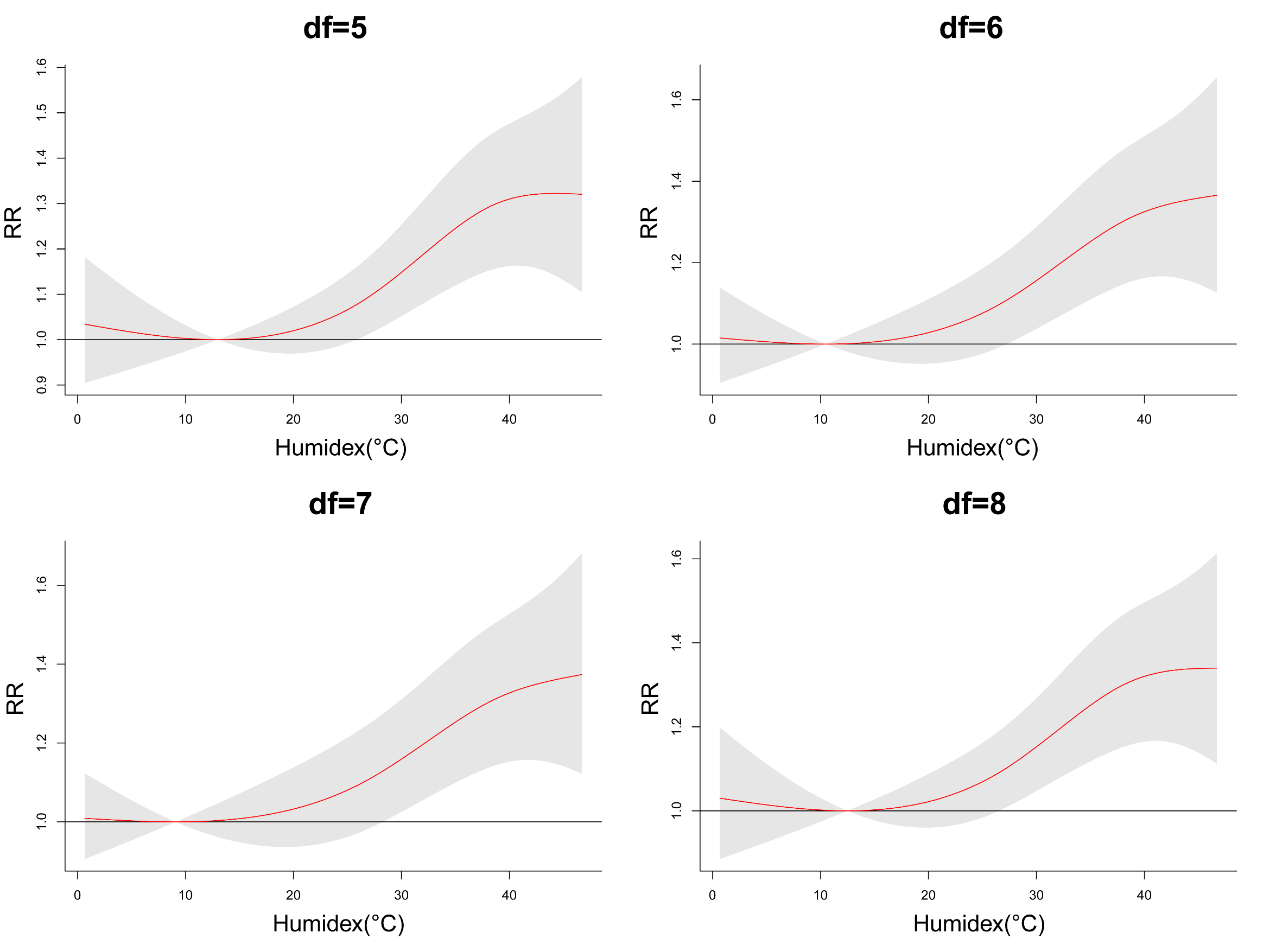


**Fig. S10.** Sensitivity analysis when altering the degrees of freedom (*df* = 5–8) for long-term trend (lag 0–5).

Abbreviations: Humidex, humidity index.


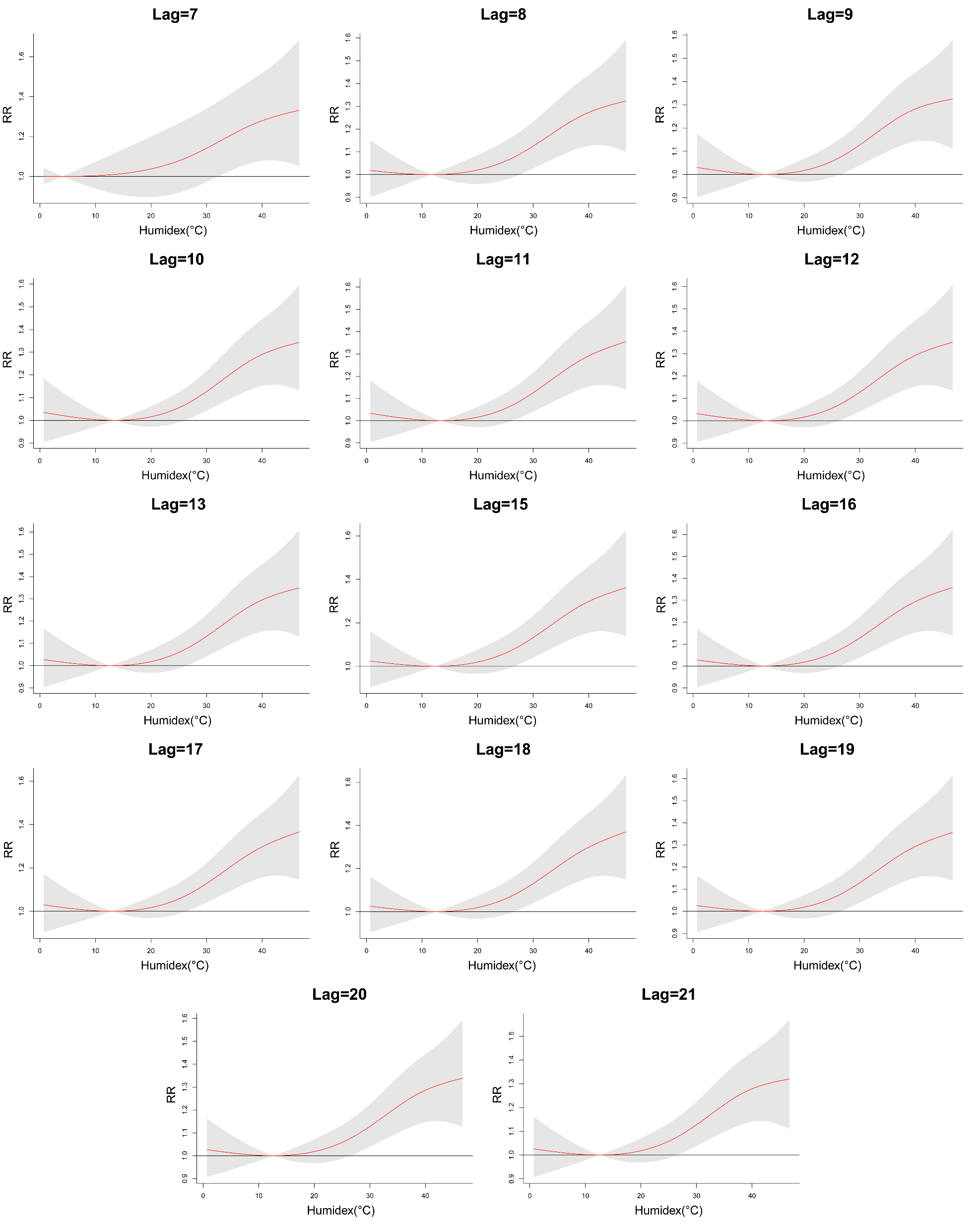


**Fig. S11.** Sensitivity analysis when altering the maximum lag days (lag= 7–13, 15–21) for cross-basis function (lag 0–5).

Abbreviations: Humidex, humidity index.


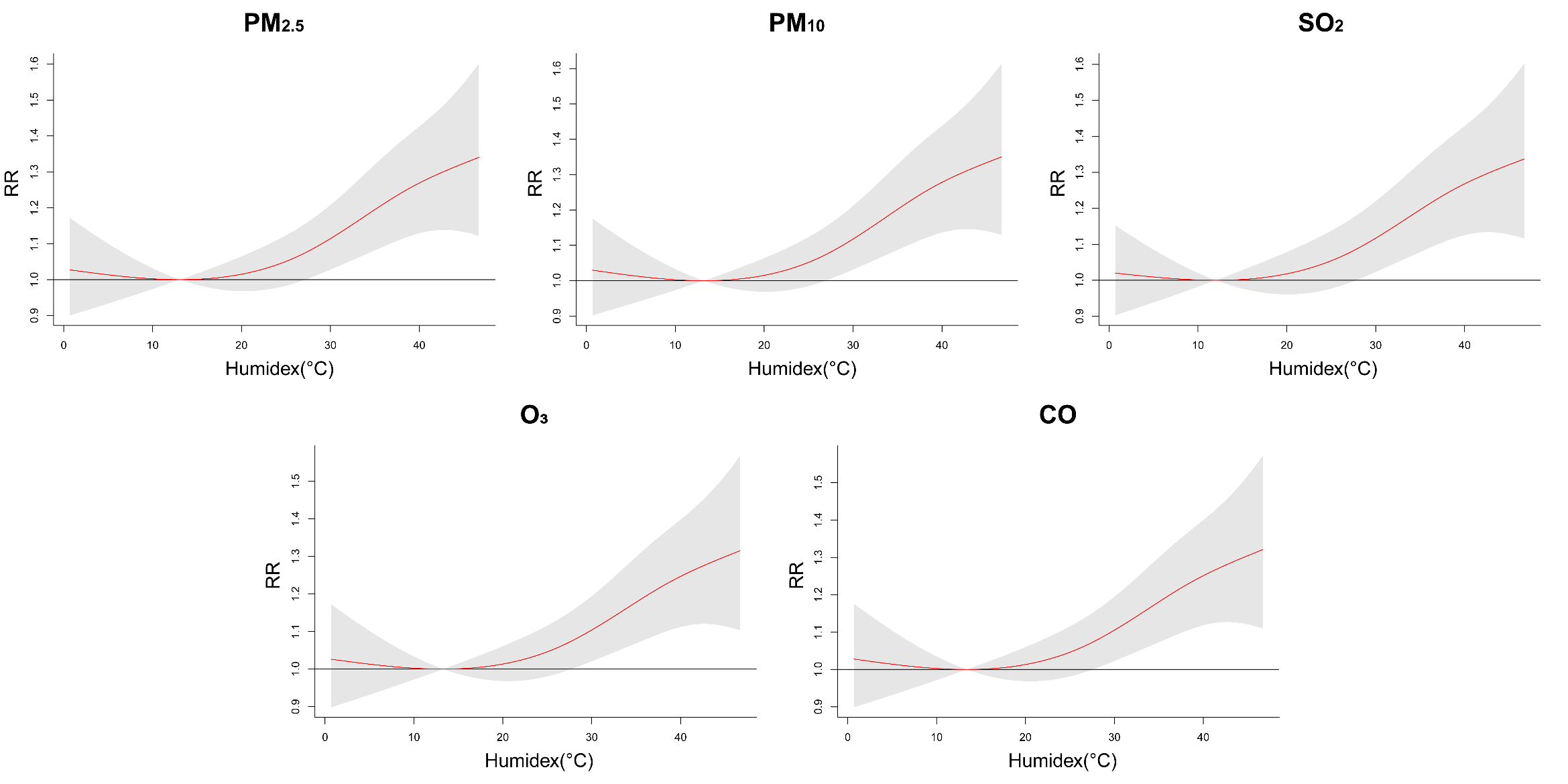


**Fig. S12.** Sensitivity analysis when replacing NO_2_ confounding factors with other pollutants confounding factors (lag 0–5).

Abbreviations: Humidex, humidity index; PM_2.5_, particulate matter less than 2.5mm in aerodynamic diameter; PM_10_, particulate matter less than 10mm in aerodynamic diameter; SO_2_, sulfur dioxide; O_3_, ozone; CO, carbon monoxide.


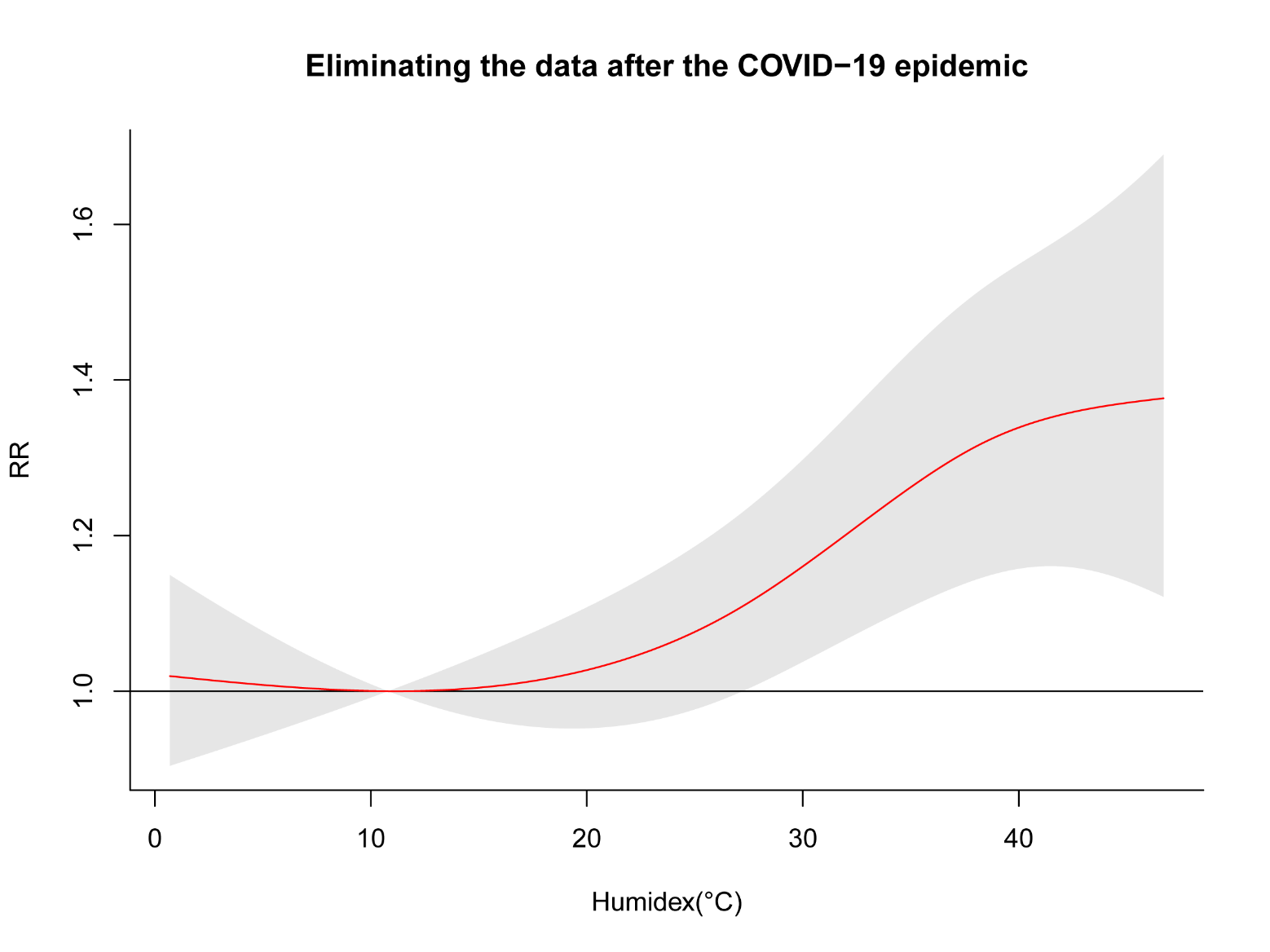


**Fig. S13.** Sensitivity analysis when eliminating the data after the COVID-19 epidemic (lag 0–5).

Abbreviations: Humidex, humidity index.


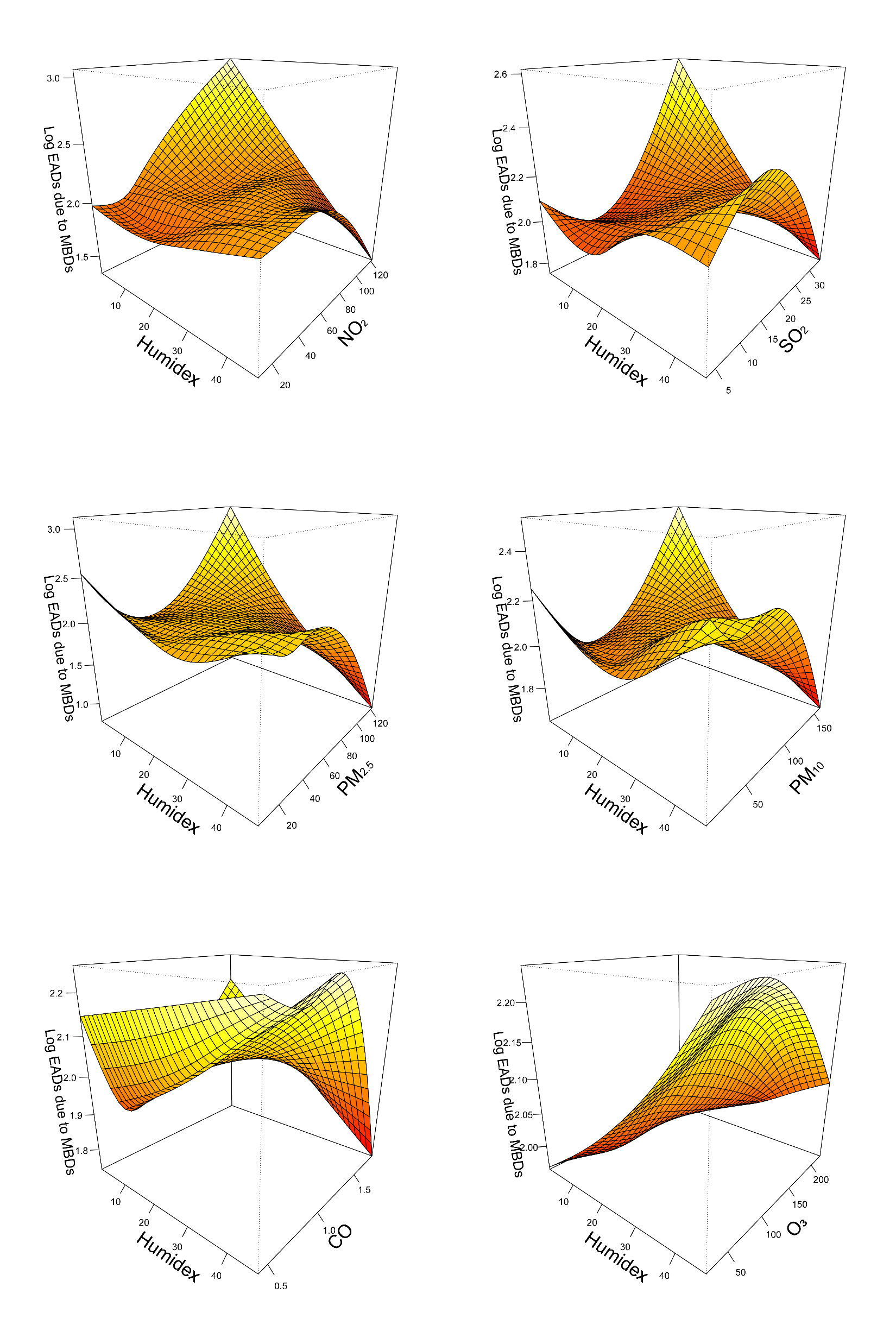


**Fig. S14.** 3D graph illustrating the interaction effect of air pollutants and the Humidex on EADs due to MBDs.

Abbreviations: Humidex, humidity index; PM_2.5_, particulate matter less than 2.5mm in aerodynamic diameter; PM_10_, particulate matter less than 10mm in aerodynamic diameter; NO_2_, nitrogen dioxide; SO_2_, sulfur dioxide; O_3_, ozone; CO, carbon monoxide.
